# Crypt-Level Tight Junction Remodeling Is Associated with Disease Course and Clinical Outcomes in Inflammatory Bowel Disease

**DOI:** 10.3390/cells15080695

**Published:** 2026-04-15

**Authors:** Efthymios P. Tsounis, Christina Geramoutsou, Ploutarchos Pastras, Ioanna Aggeletopoulou, Pinelopi Bosgana, Theoni Lourida, Georgia Diamantopoulou, Sofia Ritsatou, Efthymios Koniaris, Gerassimos J. Mantzaris, Vasiliki Zolota, Stelios F. Assimakopoulos, Vasiliki Bravou, Konstantinos Thomopoulos, Georgios Theocharis, Christos Triantos

**Affiliations:** 1 Division of Gastroenterology, Department of Internal Medicine, University of Patras, 26504 Patras, Greece; makotsouno@gmail.com (E.P.T.); ploutarchosp96@gmail.com (P.P.); iaggel@upatras.gr (I.A.); theoni_25@hotmail.com (T.L.); geodiamant@hotmail.com (G.D.); kxthomo@hotmail.com (K.T.); giorgistheocharis@gmail.com (G.T.); 2 Department of Anatomy-Histology-Embryology, Medical School, University of Patras, 26504 Patras, Greece; chrisgeram@gmail.com (C.G.); vibra@upatras.gr (V.B.); 3 Department of Pathology, School of Medicine, University of Patras, 26504 Patras, Greece; bosgana.p@gmail.com (P.B.); zol@med.upatras.gr (V.Z.); 4 Department of Pathology-Anatomy, Hippocration Hospital of Athens, Athens Medical School, National and Kapodistrian University of Athens, 11528 Athens, Greece; sofritsy@yahoo.gr (S.R.); e.koniaris@hippocratio.gr (E.K.); 5 Department of Gastroenterology, White Cross Hospital, 11528 Athens, Greece; gjmantzaris@gmail.com; 6 Department of Gastroenterology, HYGEIA Hospital, 15123 Athens, Greece; 7 Division of Infectious Diseases, Department of Internal Medicine, University Hospital of Patras, 26504 Patras, Greece; sassim@upatras.gr

**Keywords:** tight junction, inflammatory bowel disease, Crohn’s disease, ulcerative colitis, occludin, claudin-1, intestinal barrier, barrier dysfunction, clinical outcomes

## Abstract

**Highlights:**

**What are the main findings?**

- Tight junction proteins occludin and claudin-1 show increased expression and aberrant cytoplasmic localization in active inflammatory bowel disease, particularly within the crypt epithelium.
- Crypt-level occludin dysregulation is associated with adverse clinical outcomes, including IBD-related hospitalization, surgery, and clinical relapse.

**What are the implications of the main findings?**

- Tight junction remodeling reflects a dynamic component of epithelial barrier dysfunction associated with disease activity and progression in IBD.
- Assessment of crypt epithelial tight junction architecture may provide a novel tissue biomarker for risk stratification and therapeutic response monitoring.

**Abstract:**

Background: Intestinal barrier dysfunction is a hallmark of inflammatory bowel disease (IBD), yet the clinical significance of tight junction (TJ) remodeling remains unclear. We investigated whether alterations in the expression and localization of key TJ proteins are associated with disease activity and clinical outcomes in IBD. Methods: This retrospective, single-center study included patients with Crohn’s disease (CD; *n* = 100), ulcerative colitis (UC; *n* = 120), and healthy controls (*n* = 80). Immunohistochemistry was used to assess the expression and subcellular localization of occludin and claudin-1 separately in surface (SE) and crypt epithelium (CR), with staining classified as predominantly membranous (regular) or cytoplasmic (irregular). The primary endpoint was IBD-related hospitalization. Secondary endpoints included surgery, initiation of biologic therapy, and clinical relapse. Logistic and Cox regression models were applied, and longitudinal changes were assessed in paired biopsies. Results: Both occludin and claudin-1 were dysregulated in active disease, showing increased expression and cytoplasmic redistribution compared with remission and controls. TJ alterations were more pronounced in the CR and correlated with clinical, endoscopic, and histological activity. In CD, occludin CR overexpression was independently associated with hospitalization (aOR 1.010; *p* = 0.05) and surgery (aHR 1.013; *p* = 0.005), while irregular occludin CR staining was associated with initiation of biologic therapy (aOR 3.48; *p* = 0.03). In UC, increased occludin CR levels and irregular CR staining were associated with IBD-related hospitalization in multivariable analyses (aOR 1.014; *p* = 0.035 and aOR 2.78; *p* = 0.032, respectively). Higher occludin CR levels identified UC patients at increased risk of clinical relapse (aHR 1.012; *p* = 0.002). In paired biopsies (*n* = 127), TJ architecture—particularly in the CR—improved over time, with reduced expression and a shift toward membranous localization, most prominently in bio-experienced patients. Conclusions: TJ remodeling, particularly crypt-level occludin dysregulation, is associated with disease activity and clinical outcomes, capturing a clinically relevant dimension of epithelial barrier dysfunction in IBD.

## 1. Introduction

Inflammatory Bowel Disease (IBD) is characterized by chronic inflammation of the gastrointestinal tract, resulting from a complex interplay between genetic, microbial, environmental, and immune factors. One of the hallmarks of IBD pathogenesis is disruption of the intestinal barrier, a dynamic structure that serves as the interface between the host and the external environment [1]. The gut barrier comprises several interdependent components: the mucus layer, which provides a physical and biochemical shield against luminal antigens; the epithelial layer, which maintains structural stability and regulates selective permeability; the immune barrier, consisting of intraepithelial lymphocytes and lamina propria immune cells responsible for immune surveillance; and the commensal microbiota, which contributes to mucosal homeostasis and immune tolerance [2]. Compromise of the intestinal barrier leads to increased permeability, also referred to as a “leaky gut,” allowing for the translocation of microbial products, dietary antigens, and other xenobiotics into the lamina propria. This aberrant exposure triggers excessive activation of both innate and adaptive immune responses, perpetuating local tissue injury and systemic inflammation [3].

The epithelial monolayer is reinforced by a network of intercellular junctions, including adherens junctions and desmosomes, which provide mechanical stability, as well as gap junctions, which facilitate intercellular communication. Tight junctions (TJs), however, are the principal regulators of the paracellular pathway [4]. Located at the apical surface of epithelial cells, TJs form a dynamic seal that controls the passage of ions, solutes, and macromolecules while preventing the influx of microbial and antigenic molecules. TJs are formed by transmembrane proteins such as occludin, claudins, and junctional adhesion molecules (JAMs), which are anchored to the actin cytoskeleton through adaptor proteins including zonula occludens-1 (ZO-1) [5].

Disruption of TJ integrity has been consistently implicated in the pathogenesis of IBD; however, the precise clinical significance of these alterations remains incompletely understood. Multiple studies have demonstrated altered expression or mislocalization of key TJ proteins, including occludin and ZO-1, in active disease, correlating with increased intestinal permeability and mucosal inflammation [6]. Claudin dysregulation has also been reported, with increased claudin-1 and pore-forming claudin-2 promoting paracellular permeability. Conversely, several sealing claudins (e.g., claudin-3, -4, -5, and -8) appear to be downregulated or mislocalized [7]. This imbalance is largely driven by the pro-inflammatory cytokine environment in IBD [8]. In particular, tumor necrosis factor-α (TNF-α), a central mediator of inflammation, interferes with TJ assembly by inducing occludin endocytosis, while interleukin (IL)-6 increases TJ permeability through upregulation of claudin-2 in the intestinal epithelium [9,10]. In contrast, immunoregulatory cytokines such as IL-10 appear to strengthen TJ integrity and promote barrier restoration [8]. Furthermore, IBD-associated dysbiosis contributes to barrier dysfunction through depletion of protective metabolites, such as short-chain fatty acids, and expansion of pro-inflammatory microbial communities [11].

Despite accumulating evidence linking TJ dysregulation to mucosal injury in IBD, data regarding its clinical relevance—including associations with disease activity, longitudinal behavior, therapeutic response, and disease outcomes—remain limited. In this study, we assessed the expression of two key TJ components, occludin and claudin-1, in intestinal tissue from a large cohort of patients with IBD. We examined both quantitative expression and staining patterns in distinct epithelial compartments and explored their associations with disease activity, phenotypic characteristics, and relevant clinical features. Furthermore, we evaluated the relationship between these TJ proteins and major IBD-related outcomes and investigated their longitudinal dynamics in a real-world clinical setting.

## 2. Materials and Methods

### 2.1. Study Design

This retrospective, single-center study was conducted at the Tertiary Referral IBD Unit of the Division of Gastroenterology, University Hospital of Patras. Patients with an established IBD diagnosis were consecutively identified from the institutional IBD registry. Corresponding archived colonic and ileal biopsies obtained as part of routine clinical practice between January 2010 and January 2023 were retrieved from the institutional pathology database. Inclusion criteria were a complete and traceable IBD clinical file and the availability of biopsies originating from intestinal segments with documented disease involvement. All specimens were reviewed for adequacy prior to analysis, and biopsies of insufficient quality or containing dysplastic or neoplastic lesions were excluded. Exclusion criteria applied to all participants and included alternative causes of colitis (ischemic, cytomegalovirus, microscopic, or radiation colitis) and comorbid conditions known to impair intestinal barrier function, including active malignancy, advanced chronic liver or kidney disease, uncontrolled diabetes mellitus, and HIV infection. In total, 100 patients with Crohn’s disease (CD), 120 patients with ulcerative colitis (UC), and 80 healthy controls (HC) were included in this study. Controls were consecutively recruited from individuals undergoing screening colonoscopy with normal endoscopic findings and no clinical evidence of gastrointestinal disease. Ethical approval for this study was granted by the Scientific Review Board (Re: 43/29-01-2021) and the Ethics Committee (Re: 30/14-01-2021) of the University of Patras. All study procedures adhered to the principles of the Declaration of Helsinki.

### 2.2. Immunohistochemistry and Evaluation of Protein Expression

Immunohistochemical analysis was performed on formalin-fixed, paraffin-embedded intestinal biopsy specimens. Sections (3–4 μm) were deparaffinized and rehydrated, followed by heat-induced antigen retrieval and blocking of endogenous peroxidase activity. Immunostaining was performed using a standard immunoperoxidase method with the EnVision™ FLEX+ detection system (Dako, Agilent Technologies, Glostrup, Denmark; catalog No. K8002), with chromogenic visualization. Sections were incubated with primary antibodies against occludin (rabbit monoclonal anti-occludin, ab222691, Abcam, Cambridge, UK; dilution 1:150; lot No. GR3362410-4) and claudin-1 (rabbit monoclonal anti-claudin-1, ab129119, Abcam, Cambridge, UK; dilution 1:250; lot No. GR3319888-1).

Immunostained sections were evaluated by an experienced gastrointestinal pathologist (V.B.) blinded to clinical data using light microscopy. Surface epithelium (SE) and crypt epithelium (CR) were assessed separately. Staining intensity was graded on a four-point scale (0, absent; 1, weak; 2, moderate; 3, strong), and the proportion of positively stained cells was recorded. Protein expression was semi-quantitatively assessed using the histoscore (H-score) method, calculated as follows: H-score = (1 × percentage of cells with weak staining) + (2 × percentage of cells with moderate staining) + (3 × percentage of cells with strong staining), yielding a total score ranging from 0 to 300. For each protein, membranous and cytoplasmic staining patterns were evaluated independently when present. Staining was further classified as regular or irregular; irregular staining was defined as predominant cytoplasmic localization with reduced or discontinuous membranous staining. Classification was based on the predominant epithelial staining pattern rather than focal or isolated features within individual crypts or surface areas.

### 2.3. Histological Assessment

All biopsy specimens were stained with hematoxylin and eosin and independently evaluated by an experienced gastrointestinal pathologist (P.B.) blinded to clinical data. Histological disease activity was assessed using the Nancy Index, a validated stepwise histological scoring system originally developed for ulcerative colitis and subsequently applied to both IBD entities [12,13]. Grade 4 was assigned in the presence of mucosal ulceration. In the absence of ulceration, grading was based on the severity of neutrophilic infiltration (grades 3 or 2), whereas specimens without neutrophils were graded according to the degree of chronic inflammatory infiltrate (grades 0 or 1). For each biopsy, the highest grade observed was recorded.

### 2.4. Clinical and Disease-Related Data Collection

Clinical and demographic data were extracted from the institutional IBD registry and corresponding electronic medical records. Collected variables included age at diagnosis and at biopsy, sex, family history of IBD, smoking status, presence of extraintestinal manifestations, relevant comorbidities, duration of follow-up, and detailed IBD-related treatment exposure, including corticosteroids, immunomodulators, and biologic therapy, at baseline and during follow-up. Laboratory parameters obtained at the time of biopsy or within four weeks of the corresponding endoscopic procedure, including complete blood count and routine biochemical markers, were also recorded.

Clinical disease activity was evaluated using the Crohn’s Disease Activity Index (CDAI) for patients with CD and the Full Mayo score for patients with UC, as recorded during routine clinical assessments [14,15]. Active disease was defined as CDAI > 150 for CD and a Full Mayo score > 3 or the presence of any component of the Full Mayo score (stool frequency, rectal bleeding, physician’s global assessment, or endoscopic score) ≥ 2 for UC, in accordance with commonly used clinical definitions [16,17]. Endoscopic disease activity was assessed using the Simple Endoscopic Score for Crohn’s Disease (SES-CD) and the Mayo Endoscopic Score for UC, as documented in the corresponding endoscopy reports [18]. Disease phenotype and extent were classified according to the Montreal Classification [19].

### 2.5. Outcomes and Definitions

The primary outcome was IBD-related hospitalization during follow-up, defined as any hospital admission lasting ≥ 48 h primarily attributable to an IBD flare or disease-related complications, excluding planned admissions for elective procedures, intravenous infusions, or diagnostic endoscopy. Secondary outcomes included IBD-related surgery, initiation of biologic therapy, and clinical relapse. Surgical outcomes were confirmed through review of operative reports. For analyses of biologic initiation, patients already receiving biologic treatment at baseline were excluded from the analysis. Clinical relapse in UC was defined as the reappearance or worsening of disease-related symptoms, including rectal bleeding, leading to any of the following: initiation of remission-induction therapy (including topical treatments or systemic corticosteroids), dose escalation or modification in ongoing IBD therapy, IBD-related hospitalization, or colectomy [20]. Clinical relapse in CD was defined as recurrence or worsening of disease-related symptoms requiring treatment escalation, initiation of remission-induction therapy (including systemic corticosteroids), IBD-related hospitalization, or intestinal surgery [16]. Relapses attributable to infectious gastroenteritis or non–IBD-related causes were excluded. Steroid-dependent disease was defined as the inability to taper corticosteroids below the equivalent of prednisolone 10 mg/day (or budesonide 3 mg/day) within three months without recurrence of active disease or relapse occurring within three months following steroid discontinuation. Steroid-refractory disease was defined as persistently active disease despite adequate-dose systemic corticosteroid therapy (equivalent to prednisolone up to 1 mg/kg/day) for at least four weeks, in accordance with ECCO criteria [16].

### 2.6. Sample Size Estimation

Sample size was estimated for binary logistic regression to evaluate the relationship between a continuous biomarker and IBD-related hospitalization, defined as the primary study endpoint. The effect size was specified as the odds ratio (OR) per one–standard deviation (SD) increase in the biomarker. Assuming a two-sided α of 0.05 and 80% power, sample size requirements were calculated using standard methods for testing a single regression coefficient. In the absence of prior studies providing TJ-specific effect size estimates, an OR of 1.8 per 1-SD increase was prespecified as a moderate and clinically meaningful effect size for sample size estimation, in line with the magnitude of associations reported for histological predictors of adverse outcomes in IBD [21]. Based on reported 5-year hospitalization probabilities of 29% for UC and 36% for CD, the required total sample sizes were approximately 111 and 99, respectively [22,23].

### 2.7. Statistical Analysis

Categorical variables were summarized as counts and percentages, while differences between groups were assessed using Pearson’s chi-square or Fisher’s exact test, as appropriate. Continuous variables were expressed as median and interquartile range (IQR). Comparisons between two groups were performed using the Mann–Whitney U test and across three groups using the Kruskal–Wallis test. When the Kruskal–Wallis test indicated a statistically significant difference, post hoc pairwise comparisons were conducted using the Mann–Whitney U test with Holm–Bonferroni correction. The Wilcoxon signed-rank test was used for paired data. Paired categorical variables were compared using McNemar’s test. Associations between continuous variables were evaluated using Spearman’s rank correlation coefficient (ρ).

Binary outcomes (IBD-related hospitalization and initiation of biologic therapy) were analyzed using logistic regression, while time-to-event outcomes (IBD-related surgery and clinical relapse) were analyzed using Cox proportional hazards models. IBD-related hospitalization was prespecified as the primary endpoint. All other outcomes (initiation of biologic therapy, IBD-related surgery, and clinical relapse) were considered exploratory. Variables with a *p*-value < 0.05 in univariable analyses were entered into multivariable models. To account for multiple testing across TJ variables, false discovery rate (FDR) correction (Benjamini–Hochberg) was applied in univariable analyses for each outcome. For the primary endpoint, additional sensitivity analyses were performed using clinically selected covariates entered a priori into the multivariable models. Internal validation of multivariable models was conducted using bootstrap resampling with 1000 iterations. Multicollinearity was assessed using the variance inflation factor, with values > 2.5 considered unacceptable. When multiple TJ protein variables were significant in univariable analyses, separate multivariable models were constructed to avoid collinearity. Effect estimates are reported as odds ratios (ORs) or hazard ratios (HRs) with 95% confidence intervals (CIs). For continuous variables expressed as H-scores, effect estimates are presented per 1-unit increase. To improve interpretability, selected estimates—particularly for the primary endpoint—were expressed per 25-point increase by exponentiating the regression coefficient (i.e., OR^25^). For Cox regression analyses, the proportional hazards assumption was assessed using Schoenfeld residuals and inspection of residual plots. Patients without events were censored at their last available follow-up. Kaplan–Meier curves were generated for selected variables showing significant associations with outcomes in univariable analyses, including median-based dichotomization and staining patterns, and differences between groups were assessed using the log-rank test. All analyses were two-sided, and a *p*-value < 0.05 was considered statistically significant. Statistical analyses were performed using SPSS version 26 (IBM Corp., Armonk, NY, USA), and figures were generated using GraphPad Prism 9 (GraphPad Software, San Diego, CA, USA).

## 3. Results

### 3.1. Patient Characteristics

The study population comprised 100 patients with CD and 120 patients with UC. Baseline characteristics of the IBD cohorts are summarized in Table 1.

An additional 80 HC were included for comparative analyses. Individuals undergoing screening colonoscopy were older at the time of biopsy (median age 60.5 years) than patients with IBD (median age 37.5 years for CD and 43 years for UC; *p* < 0.001), whereas sex distribution was comparable across CD, UC, and HC (male sex: 62% in CD, 62.5% in UC, and 55% in HC; *p* = 0.52).

At the time of biopsy, patients with CD and UC were comparable with respect to body mass index, routine laboratory parameters, and histological disease activity, as assessed by the Nancy Index. Active smoking and extraintestinal manifestations were more frequent in CD, whereas patients with UC were older and had a longer time from diagnosis to biopsy. Disease location and behavior in CD and disease extent and severity in UC, classified according to the Montreal system, are detailed in Table 1. During follow-up, clinical relapse, IBD-related hospitalization, and IBD-related surgery occurred more frequently in CD than in UC (Table 1). Treatment exposure at baseline and during follow-up is summarized in Supplementary Table S1.

### 3.2. Tight Junction Protein Expression According to Disease Activity

Detailed quantitative analyses of TJ protein expression are provided in Supplementary Table S2.

**Figure 1:**
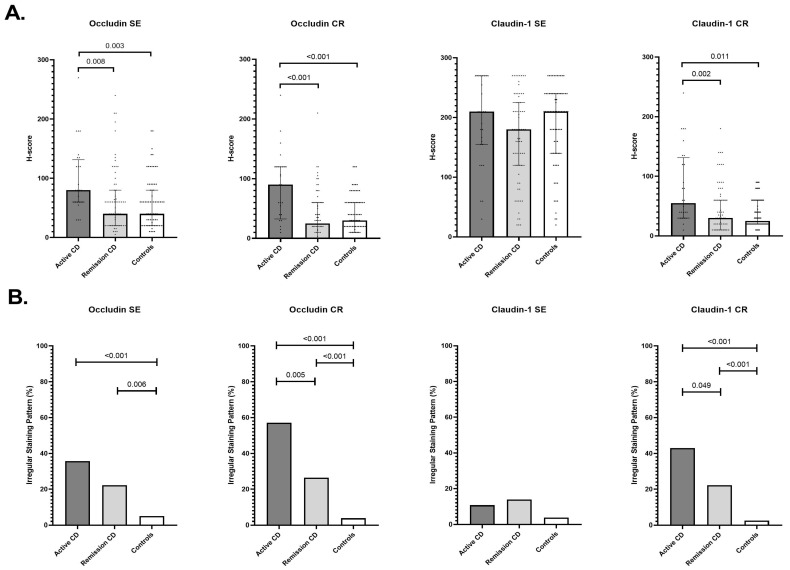
Occludin and claudin-1 expression in surface epithelium (SE) and crypt epithelium (CR) in Crohn’s disease. (**A**) H-scores for occludin and claudin-1 expression in SE and CR in patients with active Crohn’s disease (CD), CD in remission, and healthy controls. Bars represent the median and interquartile range (IQR), and dots represent individual sample values. Overall group differences were assessed using the Kruskal–Wallis test. Post hoc pairwise comparisons displayed above brackets represent Holm–Bonferroni-corrected *p*-values from Mann–Whitney U tests. (**B**) Proportion of samples with irregular occludin or claudin-1 staining in SE and CR across the same groups. Overall group comparisons were performed using the chi-square test. Pairwise comparisons displayed above brackets represent Holm–Bonferroni-corrected Fisher’s exact test *p*-values.

In CD, occludin H-scores in both SE and CR epithelium differed significantly among disease activity groups (global *p* < 0.001), with higher values in active disease compared with remission and controls (Figure 1).

Irregular occludin staining was more frequent in active CD, decreased in remission, and was rare in controls across both epithelial compartments (Supplementary Figure S1). In contrast, claudin-1 H-scores in the SE were comparable across disease activity groups, whereas CR H-scores differed significantly (global *p* = 0.006), demonstrating increased expression in active disease relative to remission and controls. Similarly, irregular claudin-1 staining in the CR showed a stepwise decline from active CD to remission and controls (Supplementary Figure S2).

**Figure 2:**
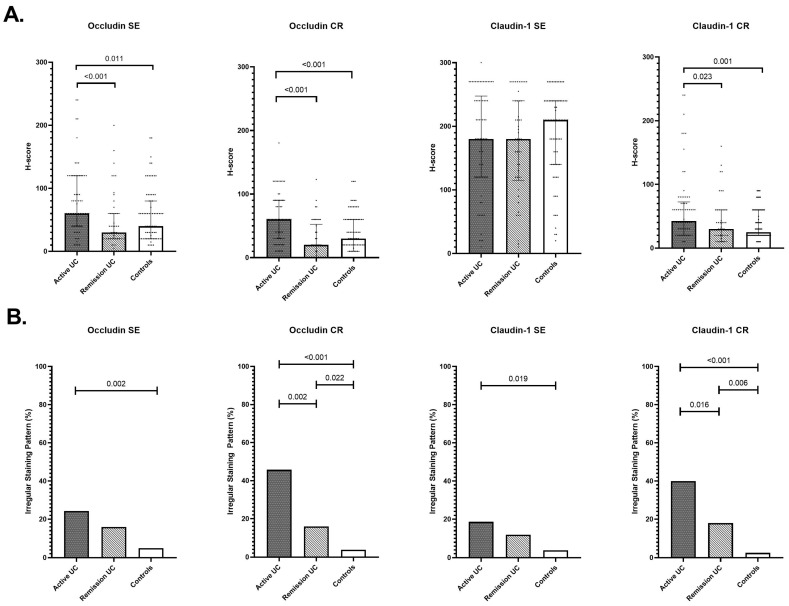
Occludin and claudin-1 expression in surface epithelium (SE) and crypt (CR) epithelium in ulcerative colitis. (**A**) H-scores for occludin and claudin-1 expression in SE and CR in patients with active ulcerative colitis (UC), UC in remission, and healthy controls. Bars represent the median with interquartile range (IQR), and dots represent individual sample values. Overall group differences were assessed using the Kruskal–Wallis test. Post hoc pairwise comparisons shown above brackets represent Holm–Bonferroni-corrected *p*-values from Mann–Whitney U tests. (**B**) Proportion of samples demonstrating irregular staining patterns of occludin or claudin-1 in SE and CR across the same groups. Overall group comparisons were performed using the chi-square test. Pairwise comparisons shown above brackets represent Holm–Bonferroni-corrected *p*-values from Fisher’s exact test.

In UC, occludin H-scores in both SE and CR epithelium also differed significantly among disease activity groups (global *p* = 0.004 and *p* < 0.001, respectively), with increased levels in active disease compared with remission and controls (Figure 2).

Irregular occludin staining was predominantly observed during active disease and was uncommon in remission and healthy controls in both epithelial compartments (Supplementary Figure S3). Claudin-1 H-scores in the SE did not differ significantly across activity groups, whereas CR H-scores showed significant variation (global *p* = 0.003), with higher levels in active disease. Irregular claudin-1 staining differed significantly across disease activity groups in both SE and CR epithelium, with the highest frequencies in active UC and the lowest in healthy controls (Supplementary Figure S4).

Direct comparisons between CD and UC revealed no significant differences in TJ protein H-scores or staining patterns in either SE or CR, both overall and after stratification by disease activity.

### 3.3. Associations Between Tight Junction Protein Expression and Disease Activity Measures

Occludin expression showed consistent associations with markers of disease activity and inflammation. Occludin CR H-scores demonstrated moderate correlations with clinical disease activity indices, including CDAI and Harvey–Bradshaw Index (HBI) in CD and the Full Mayo score in UC (ρ = 0.30–0.44; all *p* < 0.001), as well as with endoscopic scores and inflammatory markers such as CRP and platelet count (Supplementary Table S3). In addition, occludin CR expression also correlated with histological activity, as assessed by the Nancy index. Similar, but generally weaker, correlations were observed for occludin expression in the SE.

Claudin-1 SE expression showed no significant correlations with clinical or inflammatory parameters. However, claudin-1 CR expression demonstrated modest correlations with clinical and endoscopic indices, including CDAI and HBI in CD and the Full and endoscopic Mayo scores in UC (Supplementary Table S3). Correlations were also observed between occludin and claudin-1 expression across epithelial compartments, as well as between surface and crypt expression within the same protein.

### 3.4. Tight Junction Protein Expression and Clinical Outcomes in Crohn’s Disease

#### 3.4.1. IBD-Related Hospitalization in Crohn’s Disease

IBD-related hospitalization was evaluated as the primary outcome in patients with CD using logistic regression analysis. The full list of variables included in univariable models is provided in Supplementary Tables S4–S11. In univariable analyses, age, stricturing (B2) or penetrating (B3) behavior, perianal disease, and occludin expression in the CR epithelium were associated with hospitalization risk. After adjustment for multiple testing, the association for occludin CR H-score was attenuated and no longer significant (*q* = 0.072).

In multivariable models (Table 2), B2/B3 behavior (aOR 7.06, 95% CI 2.29–21.76; *p* = 0.001) remained strongly associated with hospitalization. Occludin CR H-score was also independently associated with hospitalization risk (aOR 1.010, 95% CI 1.000–1.020; *p* = 0.05). Although the per-unit effect size was small, this corresponds to an approximately 28% increase in odds per 25-point increase. Findings were consistent in bootstrap validation analyses (Supplementary Table S12).

Sensitivity analyses demonstrated a generally consistent association between occludin CR H-score and hospitalization, remaining significant after adjustment for CDAI, CRP, SES-CD, and biologic exposure, but attenuated after adjustment for histological activity (Nancy index) (Supplementary Table S13). Consistent results were observed in exploratory analyses (Supplementary Table S14) using median-based dichotomization of occludin CR expression, with higher levels associated with increased hospitalization rates (64% vs. 44%; OR 2.26; *p* = 0.046).

#### 3.4.2. Need for Biologic Therapy in Crohn’s Disease

During follow-up, initiation of biologic therapy was evaluated using logistic regression analysis. In univariable analyses, younger age, B2 or B3 disease behavior, perianal disease, and occludin parameters were associated with subsequent biologic initiation. Following adjustment for multiple testing, only irregular occludin CR staining (*q* = 0.016) and higher occludin CR H-score (*q* = 0.048) remained significant.

In multivariable models (Table 2), younger age and B2/B3 behavior remained independently associated with the need for biologic therapy across all models (age: aOR 0.95–0.96, *p* = 0.007–0.016; B2/B3 behavior: aOR 7.41–7.99, all *p* = 0.001). Irregular occludin CR staining was also independently associated with subsequent biologic therapy initiation (aOR 3.48, 95% CI 1.13–10.75; *p* = 0.03).

#### 3.4.3. IBD-Related Surgery in Crohn’s Disease

Cox proportional hazards models were used to evaluate variables associated with IBD-related surgery. In univariable analyses, B2/B3 disease behavior, perianal disease, steroid-dependent or refractory disease, higher CDAI, and certain TJ parameters were associated with an increased risk of surgery. After adjustment for multiple testing, associations for occludin CR (H-score and pattern) and claudin-1 CR H-score remained significant.

In multivariable models (Table 3), B2/B3 behavior remained the factor most strongly linked to surgery (aHR range 19.97–32.40; all *p* ≤ 0.001). Steroid-dependent or refractory disease was also consistently associated with surgery across all models (aHR range 6.17–7.34; all *p* ≤ 0.004). Furthermore, higher occludin CR H-score remained independently associated with IBD-related surgery (aHR 1.013 per unit increase; 95% CI 1.004–1.023; *p* = 0.005), together with CDAI (aHR 1.013; 95% CI 1.005–1.022; *p* = 0.002). No other TJ parameters were independently associated with surgery.

**Figure 3:**
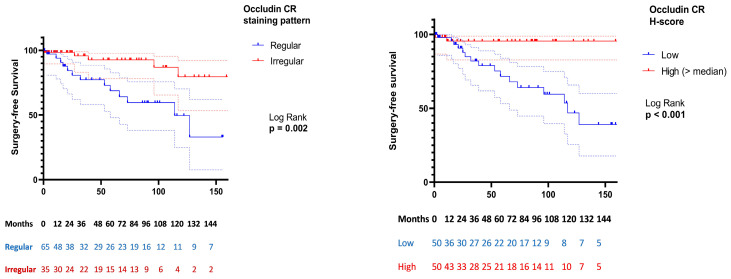
Kaplan–Meier analysis of surgery-free survival according to occludin crypt (CR) expression and staining pattern in Crohn’s disease. Surgery-free survival was defined as the time from index biopsy to first IBD-related surgery or last follow-up (censoring). Differences between groups were assessed using the log-rank test. Dashed lines represent 95% confidence intervals. Numbers at risk at predefined time points are shown below each panel.

Consistent findings were observed when occludin CR expression was analyzed categorically (median split), with higher expression accompanied by increased surgery risk (34% vs. 4%; HR 8.54; *p* = 0.004). Kaplan–Meier analysis confirmed significantly shorter surgery-free survival in patients with higher occludin CR expression compared with lower expression (mean 113.9 vs. 214.0 months; log-rank *p* = 0.001), as shown in Figure 3. Similarly, patients with irregular occludin CR staining experienced significantly shorter surgery-free survival compared with those with regular staining (mean 99.2 vs. 182.9 months; log-rank *p* = 0.002).

#### 3.4.4. Clinical Relapse in Crohn’s Disease

Factors associated with clinical relapse during follow-up were evaluated using Cox proportional hazards models. In univariable analyses, younger age, ileal or ileocolonic disease location (L1/L3), B2/B3 disease behavior, perianal disease, steroid-dependent or refractory disease, lower hemoglobin levels, and higher CDAI were associated with an increased risk of relapse.

In multivariable analysis (Table 3), B2/B3 disease behavior (aHR 2.43; 95% CI 1.34–4.42; *p* = 0.004), steroid-dependent or refractory disease (aHR 2.63; 95% CI 1.51–4.59; *p* = 0.001), and higher CDAI (aHR 1.005 per unit increase; 95% CI 1.001–1.008; *p* = 0.013) remained independently associated with relapse. None of the TJ protein parameters were significantly associated with relapse in univariable analyses.

### 3.5. Tight Junction Protein Expression and Clinical Outcomes in Ulcerative Colitis

#### 3.5.1. IBD-Related Hospitalization in Ulcerative Colitis

In univariable logistic regression analyses, moderate or severe UC (S2/S3), steroid-dependent or refractory disease, a higher Full Mayo score, increased occludin H-scores in both SE and CR epithelium, and an irregular occludin CR staining pattern were associated with hospitalization. Following accounting for multiple comparisons, all occludin parameters remained significant.

In multivariable models (Table 4), disease severity remained the strongest determinant of IBD-related hospitalization across all models (aOR range 4.53–5.43; all *p* ≤ 0.003). Higher occludin CR H-score also remained independently associated with hospitalization (aOR 1.014 per unit increase; 95% CI 1.001–1.027; *p* = 0.035), corresponding to an approximately 40% increase in odds per 25-point increase. An irregular occludin CR staining pattern was likewise independently associated with hospitalization (aOR 2.78; 95% CI 1.09–7.08; *p* = 0.032), whereas occludin SE H-score did not retain an association after adjustment. Bootstrap resampling confirmed model stability (Supplementary Table S12).

In sensitivity analyses including clinically relevant covariates (disease activity, endoscopic and histological indices, and inflammatory markers), the associations of occludin CR H-score and irregular CR staining pattern with hospitalization remained consistent, with similar effect sizes (Supplementary Table S13). Similar results were observed in exploratory analyses (Supplementary Table S14) using median-based dichotomization of occludin CR expression, with higher expression accompanied by increased hospitalization rates (43% vs. 24%; OR 2.37; *p* = 0.030).

#### 3.5.2. Need for Biologic Therapy in Ulcerative Colitis

In univariable logistic regression analyses, younger age, pancolitis (E3), moderate or severe UC (S2/S3), steroid-dependent or refractory disease, higher Full Mayo score, and an irregular occludin CR staining pattern were associated with the need for biologic therapy. After accounting for multiple testing, no TJ protein parameter remained significant.

In multivariable analysis (Table 4), disease severity (S2/S3) remained independently associated with biologic therapy initiation (aOR 3.83; 95% CI 1.12–13.11; *p* = 0.032), while no TJ protein parameter showed an independent association.

#### 3.5.3. IBD-Related Surgery in Ulcerative Colitis

No clinical or TJ protein expression variables were significantly associated with IBD-related surgery in univariable analyses in UC; multivariable analyses were therefore not performed (Table 5).

#### 3.5.4. Clinical Relapse in Ulcerative Colitis

In univariable analyses, younger age at diagnosis, moderate or severe disease (S2/S3), higher Full Mayo score, and certain occludin and claudin-1 parameters were associated with an increased risk of clinical relapse. Following FDR correction, only occludin SE (*q* = 0.004) and CR H-scores (*q* = 0.004) remained significant.

In multivariable models (Table 5), disease severity remained independently associated with clinical relapse across all models (aHR range 1.85–2.19; all *p* ≤ 0.044). In separate models including each TJ protein variable, increased occludin SE H-score (aHR 1.008; 95% CI 1.003–1.013; *p* = 0.001), increased occludin CR H-score (aHR 1.012; 95% CI 1.005–1.020; *p* = 0.002), and claudin-1 CR H-score (aHR 1.006; 95% CI 1.001–1.010; *p* = 0.019) remained independently associated with relapse.

**Figure 4:**
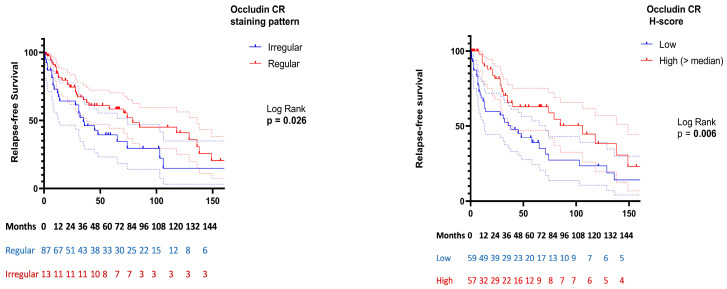
Kaplan–Meier analysis of relapse-free survival according to occludin crypt (CR) expression and staining pattern in ulcerative colitis. Relapse-free survival was defined as the time from index biopsy to first clinical relapse or last follow-up (censoring). Differences between groups were assessed using the log-rank test. Dashed lines represent 95% confidence intervals. Numbers at risk at predefined time points are shown below each panel.

In Kaplan–Meier analysis (Figure 4), regular occludin CR staining was associated with longer relapse-free survival compared with irregular staining (mean 92.3 vs. 59.2 months; log-rank *p* = 0.026). No significant differences in relapse-free survival were observed for occludin SE or claudin-1 staining patterns. When occludin CR expression was analyzed using a median split, higher expression was followed by shorter relapse-free survival (mean 61.1 vs. 97.1 months; log-rank *p* = 0.006).

### 3.6. Longitudinal Changes in Tight Junction Protein Expression in Paired Biopsies

**Figure 5:**
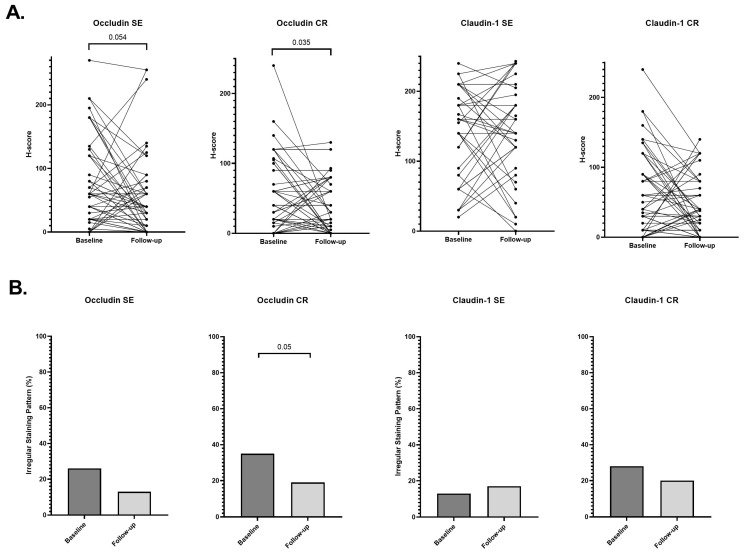
Longitudinal changes in tight junction protein expression and staining patterns in Crohn’s disease. (**A**) Paired baseline and follow-up H-scores for occludin and claudin-1 in surface epithelium (SE) and crypt epithelium (CR). Each dot represents an individual patient, with lines connecting paired samples. Paired comparisons were performed using the Wilcoxon signed-rank test. (**B**) Proportion of biopsies exhibiting irregular staining patterns at baseline and follow-up. Comparisons between time points were performed using Pearson’s chi-square test (cross-sectional analysis).

**Figure 6:**
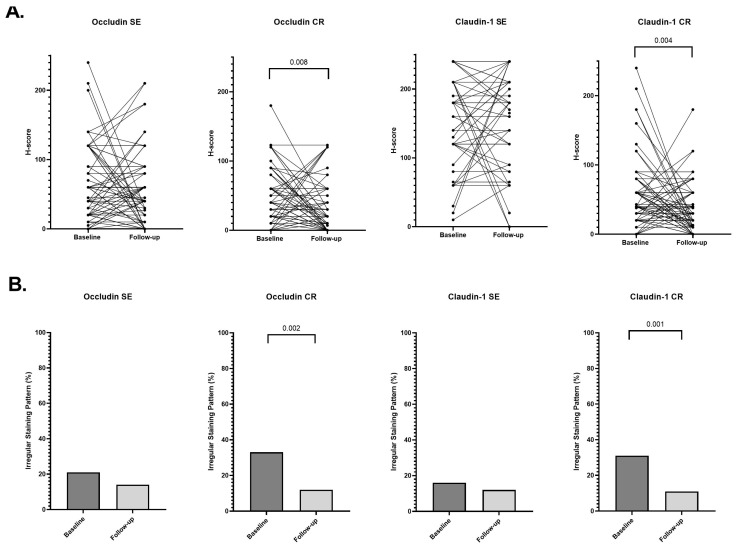
Longitudinal changes in tight junction protein expression and staining patterns in ulcerative colitis. (**A**) Paired baseline and follow-up H-scores for occludin and claudin-1 in surface epithelium (SE) and crypt epithelium (CR). Each dot represents an individual patient, with lines connecting paired samples. Paired comparisons were performed using the Wilcoxon signed-rank test. (**B**) Proportion of biopsies exhibiting irregular staining patterns at baseline and follow-up. Comparisons between time points were performed using Pearson’s chi-square test (cross-sectional analysis).

Paired analyses were performed in 127 patients with available baseline and follow-up biopsies (73 UC and 54 CD) to assess longitudinal changes in TJ protein expression. Quantitative analyses (Supplementary Table S15) demonstrated longitudinal reductions in occludin expression, most pronounced in the CR epithelium. In CD (Figure 5), occludin CR H-scores decreased from 37.5 (10–80) at baseline to 20 (0–60) at follow-up (*p* = 0.035), with a comparable reduction in UC (Figure 6; 40 [10–60] to 20 [0–50]; *p* = 0.008). A trend toward reduced occludin expression in SE was noted in CD (60 [20–90] to 40 [0–70]; *p* = 0.054) and in the pooled cohort (*p* = 0.059), but not in UC. In contrast, claudin-1 expression remained stable in SE across all analyses, whereas claudin-1 expression in CR decreased significantly in UC (40 [20–60] to 20 [10–43]; *p* = 0.004) and in the pooled cohort (*p* = 0.003), with no significant change in CD.

In parallel, the paired cohort demonstrated significant improvement in clinical and inflammatory activity over time. In CD, CDAI decreased from 111 (83–154) at baseline to 55 (36–110) at follow-up (*p* < 0.001), accompanied by a reduction in SES-CD from 6 (4–9) to 4 (2–6) (*p* < 0.001). In UC, the Full Mayo score decreased from 6 (2–8) to 2 (1–5) (*p* < 0.001), with a corresponding improvement in the Mayo endoscopic score from 2 (1–2) to 1 (1–2) (*p* < 0.001). Serum CRP levels also declined from 2.45 (0.62–5.39) to 0.44 (0.21–1.25) (*p* = 0.03). Exploratory analyses did not demonstrate a significant association between longitudinal changes in TJ expression and changes in disease activity indices (Supplementary Table S16).

Cross-sectional analyses of staining patterns (Supplementary Table S17) demonstrated a reduction in the proportion of biopsies exhibiting irregular occludin staining at follow-up, most pronounced in the CR epithelium (Figure 5 and Figure 6). This reduction was significant in UC (33% vs. 12%; *p* = 0.002) and in the pooled cohort (*p* < 0.001), with a borderline reduction in CD (35% vs. 19%; *p* = 0.050). Paired categorical analyses confirmed a significant shift toward regular occludin CR staining at follow-up in both UC (McNemar *p* = 0.008) and CD (McNemar *p* = 0.003) (Supplementary Table S18).

For claudin-1, no significant longitudinal changes were observed in SE. In contrast, irregular CR staining decreased significantly in UC (31% vs. 11%; *p* = 0.001) and in the pooled cohort (*p* = 0.003), while only a numerical reduction was observed in CD (28% vs. 20%) (Supplementary Table S17). Paired analyses likewise demonstrated a significant shift toward regular claudin-1 CR staining in UC (McNemar *p* < 0.001) and a more modest but statistically significant change in CD (McNemar *p* = 0.049) (Supplementary Table S18).

### 3.7. Effect of Biologic Therapy on Tight Junction Protein Expression

**Figure 7:**
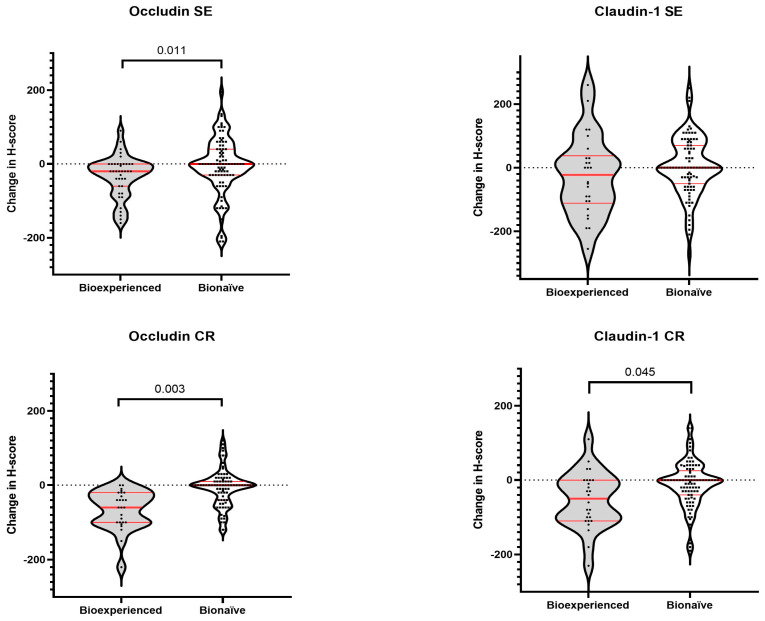
Longitudinal changes in tight junction protein expression according to biologic therapy exposure. Violin plots illustrate the distribution of changes (Δ) in H-scores for occludin and claudin-1 between baseline and follow-up, stratified by biologic therapy exposure (biologic-experienced vs. biologic-naive). Each dot represents an individual patient. Red horizontal lines indicate the median and interquartile range (IQR). Analyses are presented separately for surface epithelium (SE) and crypt epithelium (CR). Between-group comparisons were performed using the Mann–Whitney U test.

Longitudinal changes in TJ protein expression were examined according to exposure to biologic therapy (Figure 7). In the overall cohort, biologic-experienced patients exhibited greater reductions in occludin expression in both SE (median Δ = −20 vs. 0 in biologic-naive patients; *p* = 0.011) and CR epithelium (median Δ = −20 vs. 0; *p* = 0.003). A significantly greater reduction in claudin-1 expression in CR was also observed in biologic-experienced patients (median Δ = −20 vs. 0; *p* = 0.045), whereas no significant differences were detected for claudin-1 expression in SE (*p* = 0.243).

Disease-stratified analyses indicated that these associations were primarily driven by patients with CD, in whom biologic therapy was associated with larger reductions in occludin expression in SE (*p* = 0.010) and CR (*p* = 0.002). In contrast, no significant differences in longitudinal changes in occludin or claudin-1 expression were observed between biologic-experienced and biologic-naive patients with UC. Detailed results are provided in Supplementary Table S19.

## 4. Discussion

In this large, real-world cohort of patients with IBD, we demonstrate that TJ remodeling represents a key feature of disease pathophysiology potentially contributing to its natural history. The expression and epithelial localization of occludin and claudin-1—central components of TJ complexes—were markedly altered in relation to disease activity. To our knowledge, this is the first study to systematically assess surface and crypt epithelial compartments separately and to characterize subcellular localization patterns (membranous versus cytoplasmic), enabling a compartment-specific evaluation of TJ remodeling. Notably, increased occludin expression together with aberrant cytoplasmic localization in crypt epithelium was associated with a more aggressive disease course, conferring a higher risk of IBD-related hospitalization and other adverse outcomes during follow-up. Importantly, these associations were independent of established clinical, endoscopic, and histological indices. In contrast, associations involving claudin-1 were more variable across analyses and did not demonstrate comparable robustness.

A key finding emerging from our data is that increased occludin immunoreactivity in active IBD does not necessarily correspond to preserved epithelial barrier function; instead, overexpression in the context of disrupted localization likely reflects barrier dysfunction. Under inflammatory conditions, TJ proteins can be redistributed away from the apical junctional complex—where their barrier-sealing function is exerted—and accumulate intracellularly [24]. Mechanistic studies have demonstrated that pro-inflammatory cytokines, particularly TNF-α, activate MLCK-dependent signaling pathways that promote caveolin-mediated occludin internalization and TJ disassembly [9,25]. Indeed, cytokine-driven redistribution of TJ proteins within the intracellular compartment has been demonstrated in human IBD tissue [26,27]. This mechanistic framework closely mirrors the irregular occludin staining pattern observed in our cohort, with discontinuous membranous localization and prominent cytoplasmic redistribution. In this setting, occludin overexpression likely reflects a compensatory, yet ineffective, epithelial response to inflammatory injury [28]. Importantly, this irregular pattern was enriched in active disease and consistently associated with adverse clinical outcomes, whereas a predominantly membranous pattern was linked to a more favorable course. These associations were strongest within the crypt epithelium, highlighting the importance of compartment-specific TJ remodeling within the regenerative niche.

The crypt epithelium constitutes the regenerative compartment of the intestinal mucosa, housing stem and progenitor cells responsible for epithelial renewal [29]. Disruption of TJ organization at this level may have sustained downstream effects on epithelial integrity, impairing barrier function in newly generated epithelial cells and leading to persistent permeability defects even when inflammation appears attenuated [30]. This interpretation is supported by recent evidence demonstrating that epithelial abnormalities can persist despite clinical or endoscopic remission and are associated with subsequent relapse and adverse outcomes in IBD [31,32]. In our cohort, conventional histological activity (Nancy index) did not independently predict long-term outcomes, whereas crypt-level TJ remodeling remained associated with clinical outcomes in multivariable analyses. These findings suggest that barrier alterations within the regenerative epithelial compartment capture aspects of disease biology not reflected by conventional indices and may help explain why endoscopic healing does not always translate into durable remission [33,34].

Claudin-1 exhibited dynamic, inflammation-associated changes in expression and localization, particularly within the crypts, consistent with earlier studies reporting claudin-1 upregulation in inflamed IBD mucosa [35,36]. Nevertheless, these associations were less consistent and attenuated after adjustment, suggesting that claudin-1 primarily reflects acute, inflammation-driven TJ remodeling rather than persistent barrier dysfunction linked to adverse outcomes. Mechanistically, increased claudin-1 expression does not indicate preserved barrier function. Experimental studies demonstrate that claudin-1 overexpression activates ERK/MMP-9–dependent Notch signaling, suppresses goblet-cell differentiation, and reduces MUC2 expression, thereby weakening the mucus barrier and facilitating microbial translocation [37]. In addition, claudin-1 has been implicated in β-catenin and AKT-dependent signaling pathways, supporting its role in maladaptive epithelial responses under inflammatory stress [38]. Emerging data further link claudin-1 dysregulation to host–microbiome interactions: depletion of the butyrate-producing genus *Butyricicoccus* correlates inversely with claudin-1 expression in active UC, while butyrate supplementation or *Butyricicoccus pullicaecorum*-conditioned media restore claudin-1 levels in inflamed mucosa [39]. These findings suggest that claudin-1 reflects context-dependent epithelial responses to inflammation and microbial signals and should be interpreted cautiously in relation to clinical outcomes.

A major strength of the present study is the integration of longitudinal analyses using paired biopsies, enabling within-patient assessment of TJ remodeling over time. By combining longitudinal evaluation with compartment-specific analysis (crypt versus surface epithelium) and detailed assessment of subcellular localization (membranous versus cytoplasmic), our approach allowed for a more nuanced characterization of epithelial barrier remodeling than previously reported in IBD. In follow-up samples, we observed a significant reduction in occludin and claudin-1 expression accompanied by a shift toward membranous localization, most consistently within the crypts. These changes were particularly pronounced in patients receiving biologic therapy and in those with CD.

Importantly, improvement in TJ protein expression patterns in patients undergoing effective therapy supports a potential link between inflammatory control and restoration of barrier function, in line with prior studies demonstrating improved gut barrier function following successful anti-TNF treatment [40,41]. These longitudinal changes should be interpreted with caution, as they likely reflect, at least in part, reduced inflammatory activity. However, our exploratory analysis showed that changes in TJ expression did not parallel reductions in disease activity, suggesting that TJ remodeling is not fully explained by resolution of inflammation alone. Collectively, these findings highlight epithelial barrier remodeling as an under-recognized component of therapeutic response in IBD and introduce crypt-level TJ organization as a candidate marker of tissue-level response to treatment.

From a clinical and translational perspective, our findings suggest that assessment of epithelial barrier architecture—particularly crypt-level TJ organization—may provide information beyond conventional disease activity measures [42]. The observation that TJ remodeling remained independently related to clinical outcomes, while standard histological indices did not, highlights the potential value of barrier-focused biomarkers in identifying patients at risk of adverse outcomes despite apparent mucosal healing [31,43]. Moreover, the shift toward a more normal TJ architecture observed in patients responding to biologic therapy indicates that gut barrier features may serve as complementary markers of treatment response and epithelial recovery [44]. A comparable barrier-centered framework has been explored in celiac disease, where gliadin-induced zonulin signaling disrupts TJ integrity and increases permeability [45]. In this context, larazotide acetate, a zonulin antagonist, has been evaluated in randomized trials, providing proof-of-concept that pharmacologic modulation of TJ is feasible in immune-mediated enteropathies [46].

Future prospective studies are warranted to determine whether epithelial barrier parameters can be standardized and integrated into risk stratification or treatment-response frameworks and to assess the therapeutic potential of targeting crypt-level TJ integrity in IBD.

Several limitations should be acknowledged. First, the observational, retrospective design precludes causal inference between TJ remodeling and clinical outcomes. In addition, this was a single-center study conducted in a tertiary referral setting, which may introduce selection bias and limit generalizability. Follow-up biopsies were obtained as part of routine clinical care rather than at standardized time points, introducing heterogeneity in biopsy timing and treatment exposure. Second, although immunohistochemical evaluation was performed by an experienced gastrointestinal pathologist blinded to clinical data, assessments were not independently repeated by a second observer, and interobserver variability could not be formally evaluated. However, the use of predefined epithelial compartments, standardized scoring criteria, and blinded assessment was intended to minimize subjectivity. Third, functional measures of paracellular permeability were not available, and structural alterations could therefore not be directly linked to barrier function; nevertheless, the consistent associations with hard clinical endpoints support the biological relevance of the observed findings. Finally, healthy controls were not formally matched for age, sex, or biopsy site, which may have introduced confounding in comparative analyses, and no external validation cohort was available. Accordingly, these findings should be considered hypothesis-generating and require validation in prospective, multicenter studies.

## 5. Conclusions

In conclusion, this study identifies TJ remodeling—particularly aberrant occludin expression within the crypt epithelium—as a dynamic and clinically significant feature of IBD. The association of crypt-level occludin abnormalities with disease course and therapeutic response, independent of established clinical and histological measures, underscores their relevance beyond conventional tissue assessment. These findings support further exploration of TJ parameters as tissue-based biomarkers of disease behavior and treatment effectiveness, with implications for risk stratification and longitudinal monitoring. They also suggest that improvement in crypt-level TJ architecture may represent a component of deeper tissue healing.

## Figures and Tables

**Table 1:** Baseline demographic, clinical, laboratory, and disease-related characteristics of patients with Crohn’s disease and ulcerative colitis.

	Crohn’s Disease (*n* = 100)	Ulcerative Colitis (*n* = 120)	
	Median (IQR)	Median (IQR)	*p-Value*
Age, years	37.5 (27–53)	43 (31–58)	**0.035**
Body mass index, kg/m^2^	24.2 (21–29)	24.2 (22.3–27.5)	0.938
Age at diagnosis, years	35 (24–51)	39 (27–48)	0.269
Time from diagnosis to biopsy, months	0 (0–58)	14 (0–95)	**0.007**
Hemoglobin, g/dL	12.9 (11.6–14.2)	12.8 (11–14.2)	0.603
White blood cells, ×10^9^/L	8.3 (6.3–11)	8.0 (6.3–10.4)	0.663
Platelets, ×10^9^/L	307 (241–415)	277 (223–367)	0.193
CRP, mg/L	1.4 (0.5–4)	1.4 (0.4–4.5)	0.945
Albumin, g/dL	3.9 (3.1–4.3)	3.8 (3.2–4.3)	0.696
CDAI	88 (44–152)	—	—
HBI	7 (4–10)	—	—
SES-CD	6 (4–9)	—	—
Full Mayo score	—	5.5 (2–7.75)	—
Mayo endoscopic score	—	2 (1–2)	—
Nancy Index	3 (1–3)	3 (2–3)	0.958
Follow-up duration, months	61 (20–106)	57 (28–111)	0.386
	**N (%)**	**N (%)**	***p-value***
Male sex	62 (62%)	75 (62.5%)	1.000
Active smoker	45 (45%)	22 (18.3%)	**<0.001**
Family history of IBD	7 (7%)	14 (11.7%)	0.346
Extraintestinal manifestations	33 (33%)	23 (19.2%)	**0.029**
*Montreal Classification*			
*Location (CD)*			
Terminal Ileum (L1)	27 (27%)		—
Colon (L2)	17 (17%)		
Ileo-colonic (L3)	56 (56%)		
Upper GI modifier (L4)	12 (12%)		
*Behavior (CD)*			
Non-stricturing non-penetrating (B1)	59 (59%)		—
Stricturing (B2)	26 (26%)		
Penetrating (B3)	15 (15%)		
Perianal disease modifier (p)	17 (17%)		
*Extent (UC)*			
Ulcerative proctitis (E1)		27 (22.5%)	—
Left-sided UC (E2)		40 (33.3%)	
Extensive UC (E3)		53 (44.2%)	
*Severity (UC)*			
Clinical remission (S0)		19 (15.8%)	—
Mild (S1)		47 (39.2%)	
Moderate (S2)		43 (35.8%)	
Severe (S3)		11 (9.2%)	
Relapse during follow-up	67 (67%)	62 (51.7%)	**0.031**
Steroid-dependent or refractory disease	40 (40%)	42 (35%)	0.480
IBD-related hospitalization during follow-up	54 (54%)	40 (33.3%)	**0.003**
IBD-related surgery during follow-up	19 (19%)	6 (5%)	**0.002**
IBD-related cancer during follow-up	2 (2%)	4 (3.3%)	0.850
Death during follow-up	4 (4%)	4 (3.3%)	1.000

*p*-values were calculated using the Mann–Whitney U test for continuous variables and Pearson’s χ^2^ test or Fisher’s exact test for categorical variables, as appropriate. An additional 80 healthy controls were included for comparative analyses; their age and sex distributions are reported in the text only. Bold values indicate statistical significance (*p* < 0.05).

**Table 2:** Logistic regression analyses for IBD-related hospitalization and need for biologic therapy in Crohn’s disease patients.

	IBD-RELATED HOSPITALIZATION		NEED FOR BIOLOGIC THERAPY			
Variable	Univariable OR (95% CI); *p*	Multivariable Model aOR (95% CI); *p*	Univariable OR (95% CI); *p*	Multivariable Model 1 aOR (95% CI); *p*	Multivariable Model 2 aOR (95% CI); *p*	Multivariable Model 3 aOR (95% CI); *p*
Age (years)	0.962 (0.936–0.989); **0.006**	0.960 (0.930–0.990); **0.009**	0.969 (0.941–0.997); **0.029**	0.953 (0.920–0.987); **0.007**	0.957 (0.924–0.992); **0.016**	0.955 (0.922–0.989); **0.011**
Behavior (B2/B3 vs. B1)	5.980 (2.411–14.833); **<0.001**	7.060 (2.290–21.760); **0.001**	7.125 (2.646–19.188); **<0.001**	7.994 (2.475–25.820); **0.001**	7.408 (2.283–24.044); **0.001**	7.442 (2.223–24.917); **0.001**
Perianal disease	3.329 (1.002–11.058); **0.050**	1.070 (0.240–4.860); 0.932	7.333 (1.499–35.872); **0.014**	3.841 (0.552–26.738); 0.174	3.964 (0.586–26.828); 0.158	3.719 (0.513–26.960); 0.194
Occludin SE pattern (irregular vs. regular)	1.008 (0.411–2.471); 0.985 (*q* = 0.985)	—	2.850 (1.053–7.712); **0.039** (*q* = 0.104)	2.490 (0.768–8.075); 0.129	—	—
Occludin CR (H-score)	1.013 (1.003–1.022); **0.009** (*q* = 0.072)	1.010 (1.000–1.020); **0.050**	1.013 (1.003–1.023); **0.012 (*q* = 0.048)**	—	1.009 (0.998–1.020); 0.105	—
Occludin CR pattern (irregular vs. regular)	2.102 (0.898–4.923); 0.087 (*q* = 0.348)	—	4.544 (1.745–11.831); **0.002 (*q* = 0.016)**	—	—	3.478 (1.125–10.750); **0.030**
Model accuracy (AUC, 95% CI)	—	0.80 (0.71–0.88)	—	0.81 (0.72–0.90)	0.82 (0.72–0.90)	0.84 (0.75–0.92)

Separate multivariable models were fitted, each including clinical covariables and one tight junction protein variable at a time, to avoid collinearity. *q*-values represent FDR-adjusted *p*-values. Bold values indicate statistical significance (*p* < 0.05).

**Table 3:** Cox proportional hazards regression analyses for IBD-related surgery and clinical relapse in Crohn’s disease patients.

	IBD-RELATED SURGERY					CLINICAL RELAPSE	
Variable	Univariable HR (95% CI); *p*	Multivariable Model 1 aHR (95% CI); *p*	Multivariable Model 2 aHR (95% CI); *p*	Multivariable Model 3 aHR (95% CI); *p*	Multivariable Model 4 aHR (95% CI); *p*	Univariable HR (95% CI); *p*	Multivariable aHR (95% CI); *p*
Age (years)	0.972 (0.942–1.002); 0.067	—	—	—	—	0.978 (0.961–0.995); **0.013**	0.993 (0.974–1.012); 0.471
Disease location (L1/L3 vs. L2)	2.397 (0.552–10.411); 0.244	—	—	—	—	2.464 (1.158–5.242); **0.019**	1.968 (0.876–4.421); 0.101
Behavior (B2/B3 vs. B1)	9.185 (2.652–31.807); ***p* < 0.001**	20.03 (3.17–126.47); **0.001**	20.35 (3.28–126.16); **0.001**	32.40 (5.05–207.98); ***p* < 0.001**	19.97 (3.60–110.80); **0.001**	2.807 (1.662–4.742); **<0.001**	2.431 (1.336–4.422); **0.004**
Perianal disease	3.815 (1.493–9.752); **0.005**	1.88 (0.58–6.07); 0.291	1.27 (0.42–3.83); 0.673	1.21 (0.38–3.80); 0.748	1.08 (0.34–3.43); 0.895	2.592 (1.379–4.871); **0.003**	1.770 (0.883–3.550); 0.108
Hemoglobin	0.862 (0.668–1.114); 0.257	—	—	—	—	0.824 (0.687–0.988); **0.036**	0.967 (0.814–1.149); 0.705
Steroid-dependent/refractory disease	3.466 (1.319–9.108); **0.012**	6.62 (1.90–23.04); **0.003**	6.82 (2.09–22.28); **0.001**	6.17 (1.77–21.53); **0.004**	7.34 (2.14–25.18); **0.001**	2.333 (1.367–3.982); **0.002**	2.634 (1.512–4.589); **0.001**
CDAI	1.007 (1.001–1.012); **0.022**	1.009 (1.000–1.017); 0.058	1.012 (1.004–1.019); **0.004**	1.013 (1.005–1.022); **0.002**	1.015 (1.006–1.024); **0.002**	1.004 (1.001–1.007); **0.015**	1.005 (1.001–1.008); **0.013**
Occludin CR (H-score)	1.014 (1.007–1.021); ***p* < 0.001 (*q* = 0.008)**	1.013 (1.004–1.023); **0.005**	—	—	—	1.004 (1.000–1.008); 0.081 (*q* = 0.320)	—
Occludin CR pattern (irregular vs. regular)	4.587 (1.625–12.947); **0.004 (*q* = 0.011)**	—	2.350 (0.78–7.07); 0.127	—	—	1.503 (0.898–2.515); 0.121 (*q* = 0.320)	—
Claudin-1 SE (H-score)	1.007 (1.000–1.014); **0.039** (*q* = 0.078)	—	—	1.008 (1.000–1.017); 0.064	—	1.002 (0.999–1.005); 0.200 (*q* = 0.320)	—
Claudin-1 CR (H-score)	1.010 (1.003–1.016); **0.003 (*q* = 0.011)**	—	—	—	1.011 (1.003–1.019); **0.005**	1.000 (0.996–1.005); 0.858 (*q* = 0.959)	—
C-index, 95% CI	—	0.87 (0.80–0.94)	0.87 (0.79–0.94)	0.89 (0.82–0.95)	0.89 (0.82–0.95)	—	0.73 (0.67–0.79)

Separate multivariable models were fitted, each including clinical covariables and one tight junction protein variable at a time, to avoid collinearity. *q*-values represent FDR-adjusted *p*-values. Bold values indicate statistical significance (*p* < 0.05).

**Table 4:** Logistic regression analyses for IBD-related hospitalization and need for biologic therapy in ulcerative colitis patients.

	IBD-RELATED HOSPITALIZATION				NEED FOR BIOLOGIC THERAPY	
Variable	Univariable OR (95% CI); *p*	Multivariable Model 1 aOR (95% CI); *p*	Multivariable Model 2 aOR (95% CI); *p*	Multivariable Model 3 aOR (95% CI); *p*	Univariable OR (95% CI); *p*	Multivariable aOR (95% CI); *p*
Age (years)	0.982 (0.959–1.005); 0.120	—	—	—	0.971 (0.944–0.999); **0.044**	0.980 (0.949–1.012); 0.220
Extent (E3 vs. E2/E1)	1.932 (0.896–4.166); 0.093	—	—	—	3.667 (1.406–9.559); **0.008**	2.028 (0.665–6.184); 0.214
Severity (S2/S3 vs. S0/S1)	7.000 (2.960–16.554); **<0.001**	4.763 (1.777–12.769); **0.002**	5.428 (1.957–15.052); **0.001**	4.525 (1.695–12.080); **0.003**	8.623 (2.906–25.591); **<0.001**	3.833 (1.120–13.112); **0.032**
Steroid-dependent/refractory disease	2.636 (1.196–5.812); **0.016**	1.543 (0.617–3.856); 0.354	1.610 (0.646–4.013); 0.307	1.770 (0.710–4.414); 0.220	4.105 (1.585–10.630); **0.004**	2.258 (0.753–6.775); 0.146
Full Mayo score	1.284 (1.119–1.473); **<0.001**	1.098 (0.934–1.290); 0.256	1.058 (0.886–1.263); 0.533	1.093 (0.927–1.288); 0.289	1.382 (1.154–1.654); **<0.001**	1.192 (0.966–1.473); 0.102
Occludin SE (H-score)	1.011 (1.003–1.018); **0.005 (*q* = 0.013)**	1.008 (1.000–1.017); 0.056	—	—	0.999 (0.990–1.008); 0.840 (*q* = 0.900)	—
Occludin CR (H-score)	1.016 (1.005–1.026); **0.003 (*q* = 0.012)**	—	1.014 (1.001–1.027); **0.035**	—	1.009 (0.998–1.020); 0.116 (*q* = 0.464)	—
Occludin CR pattern (irregular vs. regular)	3.548 (1.584–7.948); **0.002 (*q* = 0.012)**	—	—	2.779 (1.091–7.076); **0.032**	2.682 (1.062–6.770); **0.037** (*q* = 0.296)	1.267 (0.404–3.970); 0.685
Model accuracy (AUC, 95% CI)	*—*	0.79 (0.70–0.87)	0.80 (0.71–0.88)	0.81 (0.73–0.88)	—	0.84 (0.74–0.91)

Separate multivariable models were fitted, each including clinical covariables and one tight junction protein variable at a time, to avoid collinearity. *q*-values represent FDR-adjusted *p*-values. Bold values indicate statistical significance (*p* < 0.05).

**Table 5:** Cox proportional hazards regression analyses for IBD-related surgery and clinical relapse in ulcerative colitis patients.

	IBD-RELATED SURGERY	CLINICAL RELAPSE				
Variable	Univariable HR (95% CI); *p*	Univariable HR (95% CI); *p*	Multivariable Model 1 aHR (95% CI); *p*	Multivariable Model 2 aHR (95% CI); *p*	Multivariable Model 3 aHR (95% CI); *p*	Multivariable Model 4 aHR (95% CI); *p*
Age at diagnosis (years)	0.972 (0.914–1.032); 0.353	0.982 (0.965–0.999); **0.049**	0.983 (0.965–1.002); 0.080	0.983 (0.966–1.001); 0.065	0.986 (0.968–1.004); 0.121	0.986 (0.968–1.004); 0.128
Severity (S2/S3 vs. S0/S1)	61.590 (0.085–44,621); 0.220	2.104 (1.231–3.599); **0.007**	2.114 (1.170–3.819); **0.013**	2.193 (1.184–4.063); **0.013**	1.848 (1.017–3.359); **0.044**	1.992 (1.101–3.605); **0.023**
Full Mayo score	1.227 (0.931–1.616); 0.147	1.094 (1.008–1.187); **0.031**	1.002 (0.915–1.097); 0.968	0.952 (0.855–1.061); 0.376	1.010 (0.915–1.115); 0.840	1.025 (0.935–1.124); 0.597
Occludin SE (H-score)	1.007 (0.995–1.020); 0.265 (*q* = 0.900)	1.007 (1.003–1.012); **0.001 (*q* = 0.004)**	1.008 (1.003–1.013); **0.001**	—	—	—
Occludin CR (H-score)	1.010 (0.994–1.027); 0.222 (*q* = 0.900)	1.011 (1.004–1.017); **0.001 (*q* = 0.004)**	—	1.012 (1.005–1.020); **0.002**	—	—
Occludin CR pattern (irregular vs. regular)	2.032 (0.408–10.113); 0.386 (*q* = 0.900)	1.771 (1.061–2.954); **0.029** (*q* = 0.058)	—	—	1.570 (0.884–2.788); 0.124	—
Claudin-1 CR (H-score)	0.999 (0.983–1.015); 0.912 (*q* = 0.900)	1.005 (1.001–1.010); **0.025** (*q* = 0.058)	—	—	—	1.006 (1.001–1.010); **0.019**
C-index, 95% CI	—	—	0.65 (0.58–0.74)	0.67 (0.60–0.76)	0.63 (0.56–0.72)	0.65 (0.58–0.73)

Separate multivariable models were fitted, each including clinical covariables and one tight junction protein variable at a time, to avoid collinearity. *q*-values represent FDR-adjusted *p*-values. Bold values indicate statistical significance (*p* < 0.05).

## Data Availability

The original contributions presented in this study are included in this article/Supplementary Materials. Further inquiries can be directed to the corresponding author.

## Supplementary Materials

The following supporting information can be downloaded at: https://www.mdpi.com/article/10.3390/cells15080695/s1, Figure S1: Immunohistochemical staining of occludin in intestinal mucosa. Figure S2: Immunohistochemical staining of claudin-1 in intestinal mucosa. Figure S3: Immunohistochemical staining of occludin in intestinal mucosa in ulcerative colitis. Figure S4: Immunohistochemical staining of claudin-1 in intestinal mucosa in ulcerative colitis. Table S1: IBD-related treatment exposure at baseline and during follow-up in patients with Crohn’s disease and ulcerative colitis. Table S2: Quantitative and qualitative assessment of occludin and claudin-1 expression according to disease activity in Crohn’s disease and ulcerative colitis. Table S3: Spearman correlation analysis between tight junction protein expression and clinical, laboratory, and endoscopic variables. Table S4: Univariable and multivariable logistic regression analyses for IBD-related hospitalization during follow-up in patients with Crohn’s disease. Table S5: Univariable and multivariable logistic regression analyses for initiation of biologic therapy during follow-up in patients with Crohn’s disease. Table S6: Univariable and multivariable Cox proportional hazards regression analyses for IBD-related surgery during follow-up in patients with Crohn’s disease. Table S7: Univariable and multivariable Cox proportional hazards regression analyses for clinical relapse during follow-up in patients with Crohn’s disease. Table S8: Univariable and multivariable logistic regression analyses for IBD-related hospitalization during follow-up in patients with ulcerative colitis. Table S9: Univariable and multivariable logistic regression analyses for initiation of biologic therapy during follow-up in patients with ulcerative colitis. Table S10: Univariable and multivariable Cox proportional hazards regression analyses for IBD-related surgery during follow-up in patients with ulcerative colitis. Table S11: Univariable and multivariable Cox proportional hazards regression analyses for clinical relapse during follow-up in patients with ulcerative colitis. Table S12: Bootstrap validation of multivariable models. Table S13: Sensitivity analyses of the association between occludin crypt features and IBD-related hospitalization. Table S14: Exploratory stratified analysis of occludin CR expression and clinical outcome. Table S15: Longitudinal changes in tight junction protein expression in paired intestinal biopsies. Table S16: Multivariable linear regression analyses of factors associated with longitudinal changes in tight junction protein expression in the crypt epithelium. Table S17: Unpaired cross-sectional comparison of irregular tight junction protein staining at baseline and follow-up time points. Table S18: Paired categorical analysis of longitudinal changes in tight junction protein staining patterns. Table S19: Association between biologic therapy and longitudinal changes in tight junction protein expression.

## Abbreviations

aHR	adjusted hazard ratio
aOR	adjusted odds ratio
AUC	area under the curve
CD	Crohn’s disease
CDAI	Crohn’s Disease Activity Index
C-index	concordance index
CI	confidence interval
CR	crypt epithelium
CRP	C-reactive protein
ECCO	European Crohn’s and Colitis Organization
FDR	false discovery rate
GI	gastrointestinal
HBI	Harvey–Bradshaw Index
HC	healthy controls
H-score	histoscore
HR	hazard ratio
IBD	inflammatory bowel disease
IQR	interquartile range
JAMs	junctional adhesion molecules
OR	odds ratio
SE	surface epithelium
SES-CD	Simple Endoscopic Score for Crohn’s Disease
SD	standard deviation
TJ	tight junction
TNF-α	tumor necrosis factor alpha
UC	ulcerative colitis
ZO-1	zonula occludens-1
