# Supplementary material for: Crypt-Level Tight Junction Remodeling Is Associated with Disease Course and Clinical Outcomes in Inflammatory Bowel Disease
Source: Cells. 2026 Apr 15;15(8):695. doi: 10.3390/cells15080695 (PMC13114733; doi:10.3390/cells15080695)
Supplement: Supplementary file 1 [file cells-15-00695-s001.zip › cells-4227947-supplementary.pdf]

Supplementary Material

Supplementary Table S1. IBD-related treatment exposure at baseline and during follow-up in patients with Crohn's disease and ulcerative colitis.

	**Crohn's Disease (n = 100)**	**Ulcerative Colitis (n = 120)**
**Baseline Treatment**		
5-ASA	25 (25%)	61 (50.8%)
Immunomodulator	10 (10%)	9 (7.5%)
Steroids	9 (9%)	8 (6.7%)
Biologic	14 (14%)	11 (9.2%)
**Treatment at last follow-up**		
5-ASA	40 (40%)	102 (85%)
Steroids	9 (9%)	10 (8.3%)
Immunomodulator	18 (18%)	21 (17.5%)
Biologic	54 (54%)	35 (29.2%)
**Biologic at last follow-up**		
Infliximab	23 (23%)	17 (14.2%)
Adalimumab	14 (14%)	6 (5%)
Golinumab	0 (0%)	1 (0.8%)
Vedolizumab	6 (6%)	7 (5.8%)
Ustekinumab	10 (10%)	3 (2.5%)
Upadacitinib	1 (1%)	1 (0.8%)
**Immunomodulator at last follow-up**		
Azathioprine	11 (11%)	18 (15%)
Methotrexate	6 (6%)	3 (2.5%)
**Biologic exposure**		
≤1 biologic	30 (30%)	23 (19.2%)
≥2 biologics	24 (24%)	12 (10%)

Data are presented as n (%). Treatment categories are not mutually exclusive. Steroid-dependent or steroid-refractory disease was defined according to ECCO criteria.

Supplementary Table S2. Quantitative and qualitative assessment of occludin and claudin-1 expression according to disease activity in Crohn's disease and ulcerative colitis.

		**Active CD**	**Remission CD**	**Healthy Controls**	**Sig.**	**Active UC**	**Remission UC**	**Healthy Controls**	**Sig.**
Occludin SE	H-score (median, IQR)	80 (60-131)	40 (20-80)	40 (20-80)	**0.001**	60 (40-120)	30 (20-60)	40 (20-80)	**0.004**
	Irregular staining pattern (n, %)	10 (35.7%)	16 (22.2%)	4 (5.0%)	**<0.001**	17 (24.3%)	8 (16.0%)	4 (5.0%)	**0.003**
Occludin CR	H-score (median, IQR)	90 (33-120)	25 (0-60)	30 (10-60)	**<0.001**	60 (30-90)	20 (0-53)	30 (10-60)	**<0.001**
	Irregular staining pattern (n, %)	16 (57.1%)	19 (26.4%)	3 (3.8%)	**<0.001**	32 (45.7%)	8 (16.0%)	3 (3.8%)	**<0.001**
Claudin-1 SE	H-score (median, IQR)	210 (155-270)	180 (120-225)	210 (140-240)	0.703	180 (120-248)	180 (115-240)	210 (140-240)	0.144
	Irregular staining pattern (n, %)	3 (10.7%)	10 (13.9%)	3 (3.8%)	0.084	13 (18.6%)	6 (12.0%)	3 (3.8%)	**0.015**
Claudin-1 CR	H-score (median, IQR)	55 (30-131)	30 (10-60)	25 (20-60)	**0.006**	43 (20-73)	30 (10-60)	20 (20-60)	**0.003**
	Irregular staining pattern (n, %)	12 (42.9%)	16 (22.2%)	2 (2.5%)	**<0.001**	28 (40.0%)	9 (18.0%)	2 (2.5%)	**<0.001**

Data are presented as median (IQR) for H-scores and as number (percentage) for irregular staining patterns. Expression of occludin and claudin-1 was assessed separately in SE and CR epithelium. Comparisons across disease activity groups (active disease, remission, and healthy controls) were performed using the Kruskal–Wallis test for continuous variables and the chi-square test or Fisher's exact test for categorical variables, as appropriate. When applicable, post-hoc pairwise comparisons were conducted using the Mann–Whitney U test or Fisher's exact test with Holm–Bonferroni correction. Bold values indicate statistical significance ($p < 0.05$).

Supplementary Table S3. Spearman correlation analysis between tight junction protein expression and clinical, laboratory, and endoscopic variables.

	**Occludin SE** *(ρ; sig.)*	**Occludin CR** *(ρ; sig.)*	**Claudin-1 SE** *(ρ; sig.)*	**Claudin-1 CR** *(ρ; sig.)*
Occludin SE (H-score)	–	(0.505; <0.001)	(0.223; <0.001)	(0.335; <0.001)
Occludin CR (H-score)	(0.505; <0.001)	–	(0.198; 0.001)	(0.379; <0.001)
Claudin-1 SE (H-score)	(0.223; <0.001)	(0.198; 0.001)	–	(0.541; <0.001)
Claudin-1 CR (H-score)	(0.335; <0.001)	(0.379; <0.001)	(0.541; <0.001)	–
Age	(-0.051; 0.382)	(-0.080; 0.167)	(-0.037; 0.519)	(-0.096; 0.098)
Body Mass Index	(0.082; 0.477)	(-0.112; 0.333)	(0.115; 0.317)	(0.082; 0.479)
Hemoglobin	(-0.059; 0.528)	(-0.154; 0.095)	(0.128; 0.166)	(0.002; 0.981)
White blood cells	(0.095; 0.314)	(0.212; 0.022)	(0.089; 0.342)	(0.153; 0.102)
Platelets	(0.272; 0.004)	(0.264; 0.005)	(0.005; 0.958)	(-0.005; 0.954)
CRP	(0.302; 0.005)	(0.437; <0.001)	(-0.026; 0.811)	(0.158; 0.145)
Albumin	(-0.444; 0.003)	(-0.342; 0.025)	(-0.045; 0.773)	(-0.069; 0.660)
Nancy Index	(0.161; 0.036)	(0.280; <0.001)	(-0.059; 0.441)	(0.089; 0.249)
Time from diagnosis to biopsy	(-0.079; 0.245)	(-0.157; 0.020)	(0.125; 0.066)	(-0.031; 0.643)
Full Mayo Score	(0.338; <0.001)	(0.444; <0.001)	(0.009; 0.924)	(0.222; 0.015)
Mayo Endoscopic Score	(0.324; <0.001)	(0.448; <0.001)	(0.071; 0.441)	(0.278; 0.002)
CDAI	(0.239; 0.017)	(0.375; <0.001)	(0.128; 0.204)	(0.245; 0.014)
HBI	(0.298; 0.019)	(0.434; <0.001)	(0.056; 0.663)	(0.289; 0.022)
SES-CD	(0.235; 0.025)	(0.357; <0.001)	(-0.175; 0.096)	(0.095; 0.367)

SE, surface epithelium; CR, crypt epithelium; CRP, C-reactive protein; CDAI, Crohn's Disease Activity Index; HBI, Harvey–Bradshaw Index; SES-CD, Simple Endoscopic Score for Crohn's Disease. Values represent Spearman's correlation coefficients (ϱ) with corresponding *p*-values. Significant correlations are color-coded according to magnitude: dark green/red for $|\varrho| \geq 0.30$ ($p < 0.05$) and light green/red for $0.10 \leq |\varrho| < 0.30$ ($p < 0.05$).

Supplementary Table S4. Univariable and multivariable logistic regression analyses for IBD-related hospitalization during follow-up in patients with Crohn’s disease.

	**Univariable Analysis**	**OR (95% CI)**	**Multivariable Model 1**	**aOR (95% CI)**	**Multivariable Model 2**	**aOR (95% CI)**
Age	**0.006**	0.962 (0.936–0.989)	**0.009**	0.96 (0.93–0.99)		
Age at diagnosis	**0.009**	0.966 (0.941–0.991)			**0.011**	0.96 (0.93–0.99)
Disease duration	0.283	0.999 (0.996–1.001)				
Gender, male	0.306	0.652 (0.287–1.479)				
Location (L1+L3 vs. L2)	0.096	2.514 (0.849–7.448)				
Behavior (B2+B3 vs. B1)	**<0.001**	5.980 (2.411–14.833)	**0.001**	7.06 (2.29–21.76)	**0.001**	8.27 (2.52–27.16)
Perianal disease	**0.050**	3.329 (1.002–11.058)	0.932	1.07 (0.24–4.86)	0.88	0.89 (0.19–4.17)
Active smoker	0.778	1.121 (0.508–2.472)				
Hemoglobin	0.786	1.038 (0.791–1.364)				
Platelets	0.592	0.999 (0.995–1.003)				
Albumin	0.645	0.751 (0.221–2.546)				
CRP	0.222	0.942 (0.856–1.037)				
Biologic exposure	0.167	2.386 (0.695–8.199)				
Steroid-dependent/refractory disease	0.107	1.970 (0.864–4.496)				
History of surgery	0.999	Not estimable				
Nancy index	0.089	1.391 (0.951–2.034)				
CDAI	0.095	1.005 (0.999–1.011)				
SES-CD	0.098	1.081 (0.986–1.186)				
Occludin SE						
H-score (continuous)	0.611 (q=0.869)	1.002 (0.995–1.009)				
Pattern (Irregular vs. Regular)	0.985 (q=0.985)	1.008 (0.411–2.471)				
Occludin CR						
H-score (continuous)	**0.009** (q=0.072)	1.013 (1.003–1.022)	**0.05**	1.01 (1.00–1.02)	**0.036**	1.01 (1.001–1.022)
Pattern (Irregular vs. Regular)	0.087 (q=0.348)	2.102 (0.898–4.923)				
Claudin-1 SE						
H-score (continuous)	0.652 (q=0.869)	0.999 (0.994–1.004)				

Pattern (Irregular vs. Regular)	0.235 (q=0.627)	2.063 (0.625–6.816)				
Claudin-1 CR						
H-score (continuous)	0.456 (q=0.869)	1.003 (0.995–1.011)				
Pattern (Irregular vs. Regular)	0.957 (q=0.985)	1.024 (0.427–2.458)				

Separate multivariable models were constructed for age at biopsy and age at diagnosis to avoid collinearity. q-values represent FDR-adjusted p-values. Bold values indicate statistical significance ($p < 0.05$).

Supplementary Table S5. Univariable and multivariable logistic regression analyses for initiation of biologic therapy during follow-up in patients with Crohn’s disease.

	Univariable Analysis	OR (95% CI)	Multivariable Model 1	aOR (95% CI)	Multivariable Model 2	aOR (95% CI)	Multivariable Model 3	aOR (95% CI)
Age	**0.029**	0.969 (0.941–0.997)	**0.007**	0.953 (0.920–0.987)	**0.016**	0.957 (0.924–0.992)	**0.011**	0.955 (0.922–0.989)
Age at diagnosis	0.051	0.973 (0.947–1.000)						
Disease duration	0.277	0.998 (0.994–1.002)						
Gender, male	0.064	0.438 (0.183–1.050)						
Location (L1+L3 vs. L2)	0.806	1.147 (0.384–3.423)						
Behavior (B2+B3 vs. B1)	**<0.001**	7.125 (2.646–19.188)	**0.001**	7.994 (2.475–25.820)	**0.001**	7.408 (2.283–24.044)	**0.001**	7.442 (2.223–24.917)
Perianal disease	**0.014**	7.333 (1.499–35.872)	0.174	3.841 (0.552–26.738)	0.158	3.964 (0.586–26.828)	0.194	3.719 (0.513–26.960)
Active smoker	0.527	1.316 (0.563–3.077)						
Hemoglobin	0.938	1.012 (0.755–1.355)						
Platelets	0.350	1.002 (0.998–1.007)						
Albumin	0.862	1.120 (0.314–4.000)						
CRP	0.947	0.997 (0.924–1.077)						
Steroid-dependent/refractory disease	0.169	1.876 (0.765–4.602)						

Nancy index	0.065	1.486 (0.976–2.263)						
CDAI	0.116	1.005 (0.999–1.012)						
SES-CD	0.177	1.070 (0.970–1.182)						
Occludin SE								
H-score (continuous)	0.230 (q=0.368)	1.005 (0.997–1.012)						
Pattern (Irregular vs. Regular)	**0.039** (q=0.104)	2.850 (1.053–7.712)	0.129	2.490 (0.768–8.075)				
Occludin CR								
H-score (continuous)	**0.012 (q=0.048)**	1.013 (1.003–1.023)			0.105	1.009 (0.998–1.020)		
Pattern (Irregular vs. Regular)	**0.002 (q=0.016)**	4.544 (1.745–11.831)					**0.03**	3.478 (1.125–10.750)
Claudin-1 SE								
H-score (continuous)	0.404 (q=0.539)	1.002 (0.997–1.008)						
Pattern (Irregular vs. Regular)	0.717 (q=0.717)	0.796 (0.231–2.737)						
Claudin-1 CR								
H-score (continuous)	0.163 (q=0.326)	1.006 (0.998–1.015)						
Pattern (Irregular vs. Regular)	0.687 (q=0.717)	1.214 (0.473–3.120)						

Separate multivariable models were constructed for tight junction protein variables to avoid collinearity. Patients receiving biologic therapy at baseline were excluded from these analyses. q-values represent FDR-adjusted p-values. Bold values indicate statistical significance ($p < 0.05$).

Supplementary Table S6. Univariable and multivariable Cox proportional hazards regression analyses for IBD-related surgery during follow-up in patients with Crohn's disease.

	Univariable Analysis	HR (95% CI)	Multivariable Model 1	aHR (95% CI)	Multivariable Model 2	Multivariable	Multivariable Model 3	aHR (95% CI)	Multivariable Model 4	aHR (95% CI)
Age	0.067	0.972 (0.942–1.002)								
Age at diagnosis	0.154	0.979 (0.951–1.008)								
Disease duration	0.557	0.997 (0.988–1.007)								
Gender, male	0.632	0.798 (0.316–2.014)								
Location (L1+L3 vs. L2)	0.244	2.397 (0.552–10.411)								
Behavior (B2+B3 vs. B1)	**<0.001**	9.185 (2.652–31.807)	**0.001**	20.03 (3.17–126.47)	**0.001**	20.35 (3.28–126.16)	**0.001**	19.97 (3.60–110.80)	**<0.001**	32.40 (5.05–207.98)
Perianal disease	**0.005**	3.815 (1.493–9.752)	0.291	1.88 (0.58–6.07)	0.673	1.27 (0.42–3.83)	0.895	1.08 (0.34–3.43)	0.748	1.21 (0.38–3.80)
Active smoker	0.715	0.839 (0.327–2.153)								
Hemoglobin	0.257	0.862 (0.668–1.114)								
Platelets	0.993	1.000 (0.996–1.004)								
Albumin	0.126	0.527 (0.231–1.199)								
CRP	0.631	0.977 (0.889–1.074)								

Biologic exposure	0.523	1.511 (0.425–5.371)								
Steroid-dependent/refractory disease	**0.012**	3.466 (1.319–9.108)	**0.003**	6.62 (1.90–23.04)	**0.001**	6.82 (2.09–22.28)	**0.001**	7.34 (2.14–25.18)	**0.004**	6.17 (1.77–21.53)
History of IBD-related surgery	0.112	3.323 (0.754-14.64)								
Nancy index	0.870	0.961 (0.599–1.541)								
CDAI	**0.022**	1.007 (1.001–1.012)	0.058	1.009 (1.000–1.017)	**0.004**	1.012 (1.004–1.019)	**0.002**	1.015 (1.006–1.024)	**0.002**	1.013 (1.005–1.022)
SES-CD	0.169	1.063 (0.974–1.159)								
Occludin SE										
H-score (continuous)	0.252 (q=0.260)	1.004 (0.997–1.011)								
Pattern (Irregular vs. Regular)	0.260 (q=0.260)	1.731 (0.666–4.498)								
Occludin CR										
H-score (continuous)	**<0.001 (q=0.008)**	1.014 (1.007–1.021)	**0.005**	1.013 (1.004–1.023)						
Pattern (Irregular vs. Regular)	**0.004 (q=0.011)**	4.587 (1.625–12.947)			0.127	2.35 (0.78–7.07)				
Claudin-1 SE										
H-score (continuous)	**0.039** (q=0.078)	1.007 (1.000–1.014)					0.064	1.008 (1.000–1.017)		
Pattern (Irregular vs. Regular)	0.173 (q=0.231)	0.246 (0.033–1.851)								

Claudin-1 CR										
H-score (continuous)	**0.003 (q=0.011)**	1.010 (1.003–1.016)							**0.005**	1.011 (1.003–1.019)
Pattern (Irregular vs. Regular)	0.104 (q=0.166)	2.128 (0.855–5.295)								

Separate multivariable models were constructed for tight junction protein variables to avoid collinearity. q-values represent FDR-adjusted p-values. Bold values indicate statistical significance ($p < 0.05$).

Supplementary Table S7. Univariable and multivariable Cox proportional hazards regression analyses for clinical relapse during follow-up in patients with Crohn's disease.

	**Univariable Analysis**	**HR (95% CI)**	**Multivariable Model**	**aHR (95% CI)**
Age	**0.013**	0.978 (0.961–0.995)	0.471	0.993 (0.974–1.012)
Age at diagnosis	**0.028**	0.982 (0.966–0.998)		
Disease duration	0.930	1.000 (0.997–1.004)		
Gender, male	0.922	1.026 (0.619–1.699)		
Location (L1+L3 vs. L2)	**0.019**	2.464 (1.158–5.242)	0.101	1.968 (0.876–4.421)
Behavior (B2+B3 vs. B1)	**<0.001**	2.807 (1.662–4.742)	**0.004**	2.431 (1.336–4.422)
Perianal disease	**0.003**	2.592 (1.379–4.871)	0.108	1.770 (0.883–3.550)
Active smoker	0.120	1.507 (0.898–2.527)		
Hemoglobin	**0.036**	0.824 (0.687–0.988)		
Platelets	0.204	1.002 (0.999–1.004)		
Albumin	0.754	0.889 (0.425–1.857)		
CRP	0.725	0.991 (0.943–1.042)		
Biologic exposure	0.331	1.386 (0.717–2.680)		
Steroid-dependent/refractory disease	**0.002**	2.333 (1.367–3.982)	**0.001**	2.634 (1.512–4.589)
History of surgery	0.189	2.191 (0.679–7.065)		
Nancy index	0.567	1.078 (0.834–1.394)		
CDAI	**0.015**	1.004 (1.001–1.007)	**0.013**	1.005 (1.001–1.008)
SES-CD	0.101	1.047 (0.991–1.106)		
Occludin SE				
H-score (continuous)	0.855 (q=0.959)	1.000 (0.996–1.004)		
Pattern (Irregular vs. Regular)	0.959 (q=0.959)	1.014 (0.589–1.747)		
Occludin CR				
H-score (continuous)	0.081 (q=0.320)	1.004 (1.000–1.008)		
Pattern (Irregular vs. Regular)	0.121 (q=0.320)	1.503 (0.898–2.515)		
Claudin-1 SE				
H-score (continuous)	0.200 (q=0.320)	1.002 (0.999–1.005)		

Pattern (Irregular vs. Regular)	0.082 (q=0.320)	2.022 (0.916–4.461)		
Claudin-1 CR				
H-score (continuous)	0.858 (q=0.959)	1.000 (0.996–1.005)		
Pattern (Irregular vs. Regular)	0.184 (q=0.320)	1.465 (0.834–2.574)		

q-values represent FDR-adjusted p-values. Bold values indicate statistical significance ($p < 0.05$).

Supplementary Table S8. Univariable and multivariable logistic regression analyses for IBD-related hospitalization during follow-up in patients with ulcerative colitis.

	Univariable Analysis	OR (95% CI)	Multi-variable Model 1	aOR (95% CI)	Multi-variable Model 2	aOR (95% CI)	Multi-variable Model 3	aOR (95% CI)
Age	0.120	0.982 (0.959–1.005)						
Age at diagnosis	0.151	0.981 (0.956–1.007)						
Disease duration	0.673	0.999 (0.997–1.002)						
Gender, male	1.000	1.000 (0.457–2.190)						
Extent (E3 vs. E2/E1)	0.093	1.932 (0.896–4.166)						
Severity (S2/S3 vs. S0/S1)	**<0.001**	7.000 (2.96–16.554)	**0.002**	4.763 (1.777–12.769)	**0.001**	5.428 (1.957–15.052)	**0.003**	4.525 (1.695–12.08)
Active smoker	0.406	1.496 (0.578–3.871)						
Hemoglobin	0.322	0.868 (0.656–1.149)						
Platelets	0.700	0.999 (0.995–1.004)						
Albumin	0.808	0.840 (0.205–3.435)						
CRP	0.935	1.004 (0.911–1.106)						
Biologic exposure	0.376	1.762 (0.503–6.169)						
Steroid-dependent/refractory disease	**0.016**	2.636 (1.196–5.812)	0.354	1.543 (0.617–3.856)	0.307	1.610 (0.646–4.013)	0.220	1.770 (0.710–4.414)
Nancy index	0.148	1.537 (0.858–2.754)						
Mayo Full Score	**<0.001**	1.284 (1.119–1.473)	0.256	1.098 (0.934–1.290)	0.533	1.058 (0.886–1.263)	0.289	1.093 (0.927–1.288)
Occludin SE								
H-score (continuous)	**0.005 (q=0.013)**	1.011 (1.003–1.018)	0.056	1.008 (1.000–1.017)				

Pattern (Irregular vs. Regular)	0.874 (q=0.874)	1.079 (0.421–2.768)						
Occludin CR								
H-score (continuous)	**0.003 (q=0.012)**	1.016 (1.005–1.026)			**0.035**	1.014 (1.001–1.027)		
Pattern (Irregular vs. Regular)	**0.002 (q=0.012)**	3.548 (1.584–7.948)					**0.032**	2.779 (1.091–7.076)
Claudin-1 SE								
H-score (continuous)	0.172 (q=0.275)	0.997 (0.992–1.001)						
Pattern (Irregular vs. Regular)	0.379 (q=0.505)	1.568 (0.575–4.274)						
Claudin-1 CR								
H-score (continuous)	0.116 (q=0.232)	1.006 (0.999–1.014)						
Pattern (Irregular vs. Regular)	0.780 (q=0.874)	1.123 (0.497–2.542)						

Separate multivariable models were fitted, each including clinical covariables and one tight junction protein variable at a time, to avoid collinearity. q-values represent FDR-adjusted p-values. Bold values indicate statistical significance ($p < 0.05$).

Supplementary Table S9. Univariable and multivariable logistic regression analyses for initiation of biologic therapy during follow-up in patients with ulcerative colitis.

	**Univariable Analysis**	**OR (95% CI)**	**Multivariable Model**	**aOR (95% CI)**
Age	**0.044**	0.971 (0.944–0.999)	0.220	0.980 (0.949–1.012)
Age at diagnosis	0.093	0.974 (0.944–1.005)		
Disease duration	0.307	0.997 (0.992–1.003)		
Gender, male	0.926	0.957 (0.375–2.442)		
Extent (E3 vs. E2/E1)	**0.008**	3.667 (1.406–9.559)	0.214	2.028 (0.665–6.184)
Severity (S2/S3 vs. S0/S1)	**<0.001**	8.623 (2.906–25.591)	**0.032**	3.833 (1.12–13.112)
Active smoker	0.219	1.922 (0.678–5.447)		
Hemoglobin	0.433	0.867 (0.608–1.238)		
Platelets	0.928	1.000 (0.995–1.006)		
Albumin	0.346	0.436 (0.078–2.451)		
CRP	0.463	0.946 (0.816–1.097)		
Steroid-dependent/refractory disease	**0.004**	4.105 (1.585–10.63)	0.146	2.258 (0.753–6.775)
Nancy index	0.569	1.216 (0.619–2.39)		
Mayo Full Score	**<0.001**	1.382 (1.154–1.654)	0.102	1.192 (0.966–1.473)
Occludin SE				
H-score (continuous)	0.840 (q=0.900)	0.999 (0.99–1.008)		
Pattern (Irregular vs. Regular)	0.422 (q=0.900)	1.556 (0.529–4.577)		
Occludin CR				
H-score (continuous)	0.116 (q=0.464)	1.009 (0.998–1.02)		
Pattern (Irregular vs. Regular)	**0.037** (q=0.269)	2.682 (1.062–6.77)	0.685	1.267 (0.404–3.97)

Claudin-1 SE				
H-score (continuous)	0.900 (q=0.900)	1 (0.995–1.006)		
Pattern (Irregular vs. Regular)	0.620 (q=0.900)	1.335 (0.427–4.172)		
Claudin-1 CR				
H-score (continuous)	0.769 (q=0.900)	1.001 (0.992–1.011)		
Pattern (Irregular vs. Regular)	0.798 (q=0.900)	1.135 (0.432–2.981)		

q-values represent FDR-adjusted p-values. Bold values indicate statistical significance ($p < 0.05$).

Supplementary Table S10. Univariable and multivariable Cox proportional hazards regression analyses for IBD-related surgery during follow-up in patients with ulcerative colitis.

	Univariable Analysis	HR (95% CI)
Age	0.362	0.976 (0.926–1.028)
Age at diagnosis	0.353	0.972 (0.914–1.032)
Disease duration	0.779	1.001 (0.993–1.009)
Gender, male	0.785	1.267 (0.232–6.924)
Extent (E3 vs. E2/E1)	0.413	2.034 (0.372–11.139)
Severity (S2/S3 vs. S0/S1)	0.220	61.59 (0.085–44,621)
Active smoker	0.177	0.308 (0.056–1.700)
Hemoglobin	0.544	0.810 (0.410–1.600)
Platelets	0.739	1.002 (0.991–1.012)
Albumin	0.670	0.640 (0.082–4.979)
CRP	0.927	0.986 (0.737–1.321)
Biologic exposure	0.551	1.929 (0.223–16.728)
Steroid-dependent/refractory disease	0.653	1.480 (0.268–8.182)
Nancy index	0.164	2.368 (0.703–7.984)
Mayo Full Score	0.147	1.227 (0.931–1.616)
Occludin SE		
H-score (continuous)	0.265 (q=0.990)	1.007 (0.995–1.020)
Pattern (Irregular vs. Regular)	0.773 (q=0.990)	0.728 (0.085–6.261)
Occludin CR		
H-score (continuous)	0.222 (q=0.990)	1.010 (0.994–1.027)
Pattern (Irregular vs. Regular)	0.386 (q=0.990)	2.032 (0.408–10.113)
Claudin-1 SE		
H-score (continuous)	0.990 (q=0.990)	1.000 (0.990–1.010)

Pattern (Irregular vs. Regular)	0.941 (q=0.990)	0.922 (0.107–7.926)
Claudin-1 CR		
H-score (continuous)	0.912 (q=0.990)	0.999 (0.983–1.015)
Pattern (Irregular vs. Regular)	0.946 (q=0.990)	0.943 (0.173–5.151)

q-values represent FDR-adjusted p-values. Bold values indicate statistical significance ($p < 0.05$).

Supplementary Table S11. Univariable and multivariable Cox proportional hazards regression analyses for clinical relapse during follow-up in patients with ulcerative colitis.

	**Univariable Analysis**	**HR (95% CI)**	**Multi-variable Model 1**	**aHR (95% CI)**	**Multi-variable Model 2**	**aHR (95% CI)**	**Multi-variable Model 3**	**aHR (95% CI)**	**Multi-variable Model 4**	**aHR (95% CI)**
Age	0.142	0.988 (0.972–1.004)								
Age at diagnosis	**0.049**	0.982 (0.965–0.999)	0.080	0.983 (0.965–1.002)	0.065	0.983 (0.966–1.001)	0.121	0.986 (0.968–1.004)	0.128	0.986 (0.968–1.004)
Disease duration	0.09	1.002 (1–1.004)								
Gender, male	0.846	1.053 (0.624–1.776)								
Extent (E3 vs. E2/E1)	0.319	1.296 (0.778–2.16)								
Severity (S2/S3 vs. S0/S1)	**0.007**	2.104 (1.231–3.599)	**0.013**	2.114 (1.170–3.819)	**0.013**	2.193 (1.184–4.063)	**0.044**	1.848 (1.017–3.359)	**0.023**	1.992 (1.101–3.605)
Active smoker	0.343	0.736 (0.39–1.389)								
Hemoglobin	0.705	0.967 (0.814–1.149)								
Platelets	0.43	0.999 (0.996–1.002)								
Albumin	0.287	1.579 (0.682–3.657)								
CRP	0.412	1.032 (0.957–1.112)								

Biologic exposure	0.131	1.786 (0.842–3.789)								
Steroid-dependent/refractory disease	0.164	1.437 (0.863–2.393)								
Nancy index	0.199	1.267 (0.883–1.818)								
Mayo Full Score	**0.031**	1.094 (1.008–1.187)	0.968	1.002 (0.915–1.097)	0.376	0.952 (0.855–1.061)	0.840	1.010 (0.915–1.115)	0.597	1.025 (0.935–1.124)
Occludin SE										
H-score (continuous)	**0.001 (q=0.004)**	1.007 (1.003–1.012)	**0.001**	1.008 (1.003–1.013)						
Pattern (Irregular vs. Regular)	0.468 (q=0.687)	1.258 (0.676–2.340)								
Occludin CR										
H-score (continuous)	**0.001 (q=0.004)**	1.011 (1.004–1.017)			**0.002**	1.012 (1.005–1.020)				
Pattern (Irregular vs. Regular)	**0.029** (q=0.058)	1.771 (1.061–2.954)					0.124	1.570 (0.884–2.788)		
Claudin-1 SE										
H-score (continuous)	0.547 (q=0.687)	1.001 (0.998–1.004)								
Pattern (Irregular vs. Regular)	0.865 (q=0.865)	0.942 (0.477–1.864)								
Claudin-1 CR										
H-score (continuous)	**0.025** (q=0.058)	1.005 (1.001–1.010)							**0.019**	1.006 (1.001–1.010)
Pattern (Irregular vs. Regular)	0.601 (q=0.687)	1.152 (0.677–1.962)								

Separate multivariable models were fitted, each including clinical covariables and one tight junction protein variable at a time, to avoid collinearity. q-values represent FDR-adjusted p-values. Bold values indicate statistical significance ($p < 0.05$).

Supplementary Table S12. Bootstrap validation of multivariable models.

Cohort	Outcome	Variable	Original estimate (95% CI)	Bootstrap 95% CI	p-value
**Crohn's disease**	Hospitalization	Occludin CR (H-score)	1.010 (1.000–1.020)	0.999–1.023	**0.045**
**Crohn's disease**	Surgery	Occludin CR (H-score)	1.013 (1.004–1.023)	1.005–1.035	**0.003**
**Crohn's disease**	Surgery	Claudin-1 CR (H-score)	1.011 (1.003–1.019)	1.001–1.034	**0.005**
**Crohn's disease**	Biologic initiation	Occludin CR pattern (irregular vs. regular)	3.48 (1.13–10.75)	1.18–13.71	**0.018**
**Ulcerative colitis**	Hospitalization	Occludin CR (H-score)	1.014 (1.001–1.027)	1.002–1.031	**0.035**
**Ulcerative colitis**	Hospitalization	Occludin CR pattern (irregular vs. regular)	2.78 (1.09–7.08)	0.98–9.14	**0.044**
**Ulcerative colitis**	Relapse	Occludin SE (H-score)	1.008 (1.003–1.013)	1.003–1.014	**0.001**
**Ulcerative colitis**	Relapse	Occludin CR (H-score)	1.012 (1.005–1.020)	1.005–1.022	**0.002**
**Ulcerative colitis**	Relapse	Claudin-1 CR (H-score)	1.006 (1.001–1.010)	1.001–1.010	**0.007**

Bootstrap validation of multivariable models (1000 resamples). Estimates are presented as odds ratios (ORs) or hazard ratios (HRs) with 95% confidence intervals (CIs). Bootstrap confidence intervals were derived from resampled coefficients. Bold values indicate statistical significance ($p < 0.05$).

Supplementary Table S13. Sensitivity analyses of the association between occludin crypt features and IBD-related hospitalization.

Panel A. Crohn's disease – Occludin crypt H-score

Model	aOR (95% CI)	p-value
Main model*	1.010 (1.000–1.020)	**0.050**
+ Disease location (L1+L3 vs. L2)	1.010 (0.999–1.020)	0.066
+ CDAI	1.015 (1.001–1.030)	**0.035**
+ SES-CD	1.013 (1.001–1.025)	**0.034**
+ CRP	1.040 (1.000–1.080)	**0.048**
+ Nancy index	1.006 (0.993–1.018)	0.372
+ Biologic exposure	1.010 (1.001–1.021)	**0.049**

Panel B. Ulcerative colitis – Occludin crypt H-score

Model	aOR (95% CI)	p-value
Main model*	1.016 (1.004–1.028)	**0.008**
+ Age	1.016 (1.004–1.027)	**0.009**
+ Disease extent (E3 vs. E2+E1)	1.016 (1.004–1.028)	**0.009**
+ Full Mayo score	1.014 (1.001–1.027)	**0.033**
+ Endoscopic activity (Mayo subscore)	1.014 (1.001–1.027)	**0.030**
+ CRP	1.019 (1.002–1.036)	**0.026**
+ Nancy index	1.013 (0.998–1.028)	0.080
+ Biologic exposure	1.016 (1.004–1.029)	**0.009**

Panel C. Ulcerative colitis – Occludin crypt staining pattern

Model	aOR (95% CI)	p-value
Main model*	3.104 (1.28–7.52)	**0.012**
+ Age	3.139 (1.288–7.651)	**0.012**
+ Disease extent (E3 vs. E2+E1)	3.082 (1.241–7.653)	**0.015**

+ Full Mayo score	2.651 (1.052–6.682)	**0.039**
+ Endoscopic activity (Mayo subscore)	2.662 (1.057–6.705)	**0.038**
+ CRP	4.567 (1.212–17.205)	**0.025**
+ Nancy index	3.589 (1.171–11.000)	**0.025**
+ Biologic exposure	3.240 (1.302–8.060)	**0.011**

*Main model definitions:

- CD: age, disease behavior (B2+B3 vs. B1), and occludin crypt H-score.

-UC (H-score): disease severity (S2+S3 vs. S0+S1) and occludin crypt H-score.

-UC (pattern): disease severity (S2+S3 vs. S0+S1) and occludin crypt staining pattern (irregular vs. regular).

Bold values indicate statistical significance ($p < 0.05$).

Supplementary Table S14. Exploratory stratified analysis of occludin CR expression and clinical outcome.

**Cohort**	**Outcome**	**Model**	**Occludin CR group**	**Event (%)**	**Unadjusted Effect estimate (95% CI)**	**p-value**
**Crohn's disease**	Hospitalization	Logistic	Low	44% (22/50)	Reference	—
			High	64% (32/50)	OR 2.26 (1.01–5.05)	0.046
**Crohn's disease**	Biologic initiation	Logistic	Low	32% (14/44)	Reference	—
			High	62% (26/42)	OR 3.48 (1.43–8.47)	0.006
**Crohn's disease**	Surgery	Cox	Low	4% (2/50)	Reference	—
			High	34% (17/50)	HR 8.54 (1.97–37.13)	0.004

**Ulcerative colitis**	Hospitalization	Logistic	Low	24% (15/62)	Reference	—
			High	43% (25/58)	OR 2.37 (1.09–5.18)	0.030
**Ulcerative colitis**	Clinical relapse	Cox	Low	40% (25/62)	Reference	—
			High	64% (37/58)	HR 2.02 (1.20–3.40)	0.008

Exploratory stratified analyses based on median occludin crypt (CR) expression (low vs high). Effect estimates are presented as odds ratios (ORs) for logistic models and hazard ratios (HRs) for Cox models. Event rates represent the proportion of patients with events during follow-up. Analyses are unadjusted and provided for interpretability. Bold values indicate statistical significance ($p < 0.05$).

Supplementary Table S15. Longitudinal changes in tight junction protein expression in paired intestinal biopsies.

Marker / Compartment	Disease	Baseline Median (IQR)	Follow-up Median (IQR)	Wilcoxon p
**Occludin – Surface epithelium**	CD	60 (20–90)	40 (0–70)	0.054
	UC	60 (20–90)	40 (10–80)	0.403
	Pooled	60 (20–90)	40 (0–80)	0.059
**Occludin – Crypts**	CD	37.5 (10–80)	20 (0–60)	**0.035**
	UC	40 (10–60)	20 (0–50)	**0.008**
	Pooled	40 (10–70)	20 (0–55)	**0.001**
**Claudin-1 – Surface epithelium**	CD	180 (120–240)	180 (120–240)	0.843
	UC	180 (120–240)	180 (120–240)	0.663
	Pooled	180 (120–240)	180 (120–240)	0.618
**Claudin-1 – Crypts**	CD	37.5 (10–80)	30 (10–80)	0.206
	UC	40 (20–60)	20 (10–43)	**0.004**
	Pooled	40 (10–70)	25 (10–55)	**0.003**

Data are presented as median (interquartile range). Paired comparisons between baseline and follow-up biopsies were performed using the Wilcoxon signed-rank test. Bold values indicate statistical significance ($p < 0.05$).

Supplementary Table S16. Multivariable linear regression analyses of factors associated with longitudinal changes in tight junction protein expression in the crypt epithelium.

Protein / Cohort	Baseline H-score β (p-value)	Biologic therapy β (p-value)	ΔActivity β (p-value)
**Occludin (CR) – CD**	−0.62 **(<0.001)**	−0.23 (**0.020**)	0.12 (0.254)
**Occludin (CR) – UC**	−0.68 **(<0.001)**	−0.04 (0.609)	0.14 (0.127)
**Claudin-1 (CR) – CD**	−0.74 **(<0.001)**	−0.11 (0.261)	−0.09 (0.375)

**Claudin-1 (CR) – UC**	−0.82 **(<0.001)**	−0.14 (**0.038**)	0.08 (0.221)

Values represent standardized regression coefficients (β). ΔDisease activity corresponds to ΔCDAI in Crohn's disease (CD) and ΔMayo score in ulcerative colitis (UC). Δ variables represent change between baseline and follow-up. Separate multivariable models were constructed for each protein and disease cohort. Time between biopsies was included in all models but was not retained due to lack of statistical significance. Bold values indicate statistical significance ($p < 0.05$).

Supplementary Table S17. Unpaired cross-sectional comparison of irregular tight junction protein staining at baseline and follow-up time points.

Marker / Compartment	CD – BL % IRREG	CD – FUP % IRREG	p (χ^2)	UC – BL % IRREG	UC – FUP % IRREG	p (χ^2)	Pooled p (χ^2)
**Occludin – Surface epithelium**	26% (26/100)	13% (7/54)	0.094	21% (25/120)	14% (10/73)	0.291	**0.038**
**Occludin – Crypts**	35% (35/100)	19% (10/54)	0.050	33% (40/120)	12% (9/73)	**0.002**	**<0.001**
**Claudin-1 – Surface epithelium**	13% (13/100)	17% (9/54)	0.705	16% (19/120)	12% (9/73)	0.646	1.000
**Claudin-1 – Crypts**	28% (28/100)	20% (11/54)	0.347	31% (37/120)	11% (8/73)	**0.001**	**0.003**

BL, baseline; FUP, follow-up; IRREG, irregular staining pattern. Data are presented as percentages with absolute numbers in parentheses. Comparisons between baseline and follow-up time points were performed using Pearson's chi-square test or Fisher's exact test, as appropriate. The pooled p-value reflects comparison between baseline and follow-up after combining CD and UC cohorts. Bold values indicate statistical significance ($p < 0.05$).

Supplementary Table S18. Paired categorical analysis of longitudinal changes in tight junction protein staining patterns.

Marker / Compartment	Disease	Paired N	REG→REG	REG→IRREG	IRREG→REG	IRREG→IRREG	McNemar p (Disease)	McNemar p (Pooled)
**Occludin – Surface epithelium**	UC	73	55	6	8	4	0.791	0.087
	CD	54	36	3	11	4	0.057	
**Occludin – Crypts**	UC	73	42	7	22	2	**0.008**	**<0.001**
	CD	54	27	3	17	7	**0.003**	
**Claudin-1 – Surface epithelium**	UC	73	55	4	9	5	0.267	0.169
	CD	54	37	5	8	4	0.581	
**Claudin-1 – Crypts**	UC	73	45	3	20	5	**<0.001**	**<0.001**
	CD	54	30	4	13	7	**0.049**	

REG, regular staining pattern; IRREG, irregular staining pattern. Data are presented as numbers of paired biopsies transitioning between staining patterns from baseline to follow-up. McNemar's test was used for paired categorical comparisons within each disease group. Pooled p-values were calculated after combining CD and UC cohorts. Bold values indicate statistical significance ($p < 0.05$).

Supplementary Table S19. Association between biologic therapy and longitudinal changes in tight junction protein expression.

Disease group	Marker / Compartment	Biologic-naive Δ median (IQR), N	Biologic-experienced Δ median (IQR), N	p (Mann–Whitney U)
**All patients**	Occludin – Surface epithelium	0 (−30 to 40), 87	−20 (−60 to 0), 40	**0.011**
	Occludin – Crypts	0 (−40 to 10), 87	−20 (−85 to 0), 40	**0.003**
	Claudin-1 – Surface epithelium	0 (−45 to 65), 87	0 (−105 to 45), 40	0.243
	Claudin-1 – Crypts	0 (−35 to 22.5), 87	−20 (−85 to 0), 40	**0.045**
**Crohn's disease**	Occludin – Surface epithelium	−30 (−30 to 60), 34	−40 (−85 to −12.5), 20	**0.010**
	Occludin – Crypts	0 (−10 to 20), 34	−40 (−95 to 0), 20	**0.002**
	Claudin-1 – Surface epithelium	0 (−60 to 85), 34	0 (−75 to 45), 20	0.706
	Claudin-1 – Crypts	0 (−30 to 30), 34	−10 (−105 to 0), 20	0.095
**Ulcerative colitis**	Occludin – Surface epithelium	−30 (−30 to 40), 53	−10 (−20 to 0), 20	0.294
	Occludin – Crypts	0 (−40 to 0), 53	−20 (−70 to 0), 20	0.202
	Claudin-1 – Surface epithelium	0 (−30 to 60), 53	−15 (−150 to 45), 20	0.223
	Claudin-1 – Crypts	0 (−40 to 0), 53	−25 (−70 to 0), 20	0.146

Data are presented as median change (Δ) from baseline to follow-up with interquartile range (IQR). Negative Δ values indicate a reduction in protein expression over time. Numbers represent the number of patients in each group. Comparisons between biologic-naive and biologic-experienced patients were

performed using the Mann–Whitney U test. Analyses are presented for the overall cohort and stratified by disease type. Bold values indicate statistical significance ($p < 0.05$).

Supplementary Figure S1. Immunohistochemical staining of occludin in intestinal mucosa.

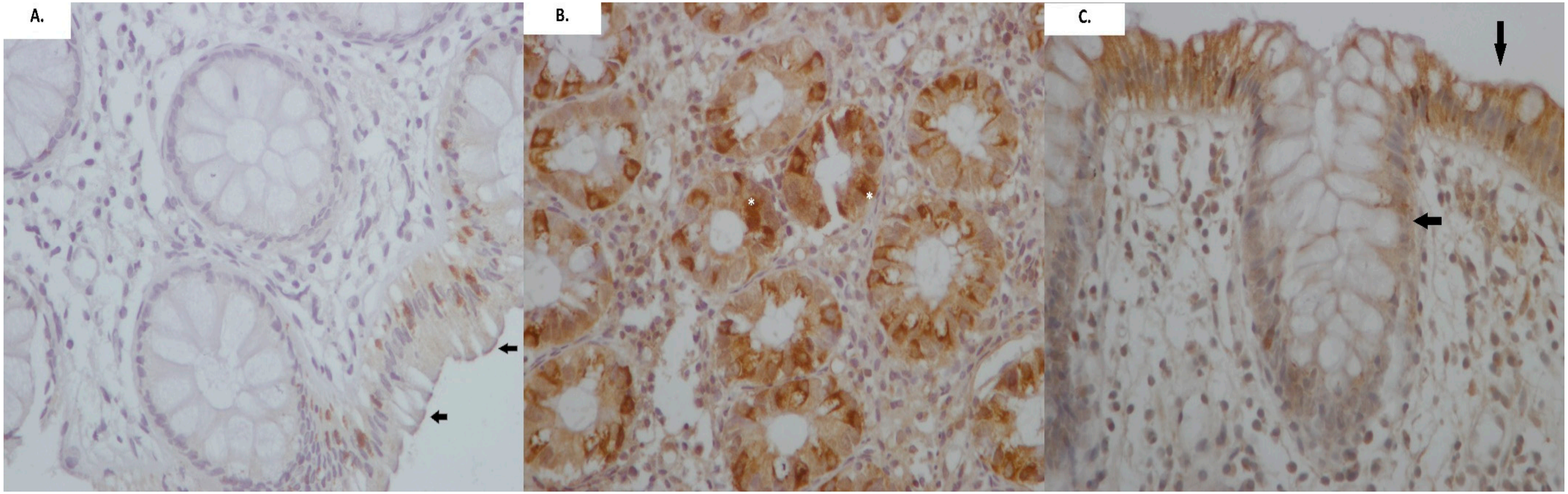

(A) Healthy control mucosa demonstrating predominantly continuous apical membranous occludin staining (regular pattern) along the luminal border of epithelial cells (arrows). (B) Active Crohn's disease showing increased occludin immunoreactivity with an aberrant pattern characterized by discontinuous membranous labeling and prominent cytoplasmic redistribution, predominantly within the crypt epithelium (asterisks). (C) Follow-up biopsy after therapy demonstrating reduced occludin expression, particularly within the crypt epithelium, together partial restoration of membranous labeling and residual cytoplasmic redistribution (arrows). Original magnification: A and C, ×400; B, ×200.

Supplementary Figure S2. Immunohistochemical staining of claudin-1 in intestinal mucosa.

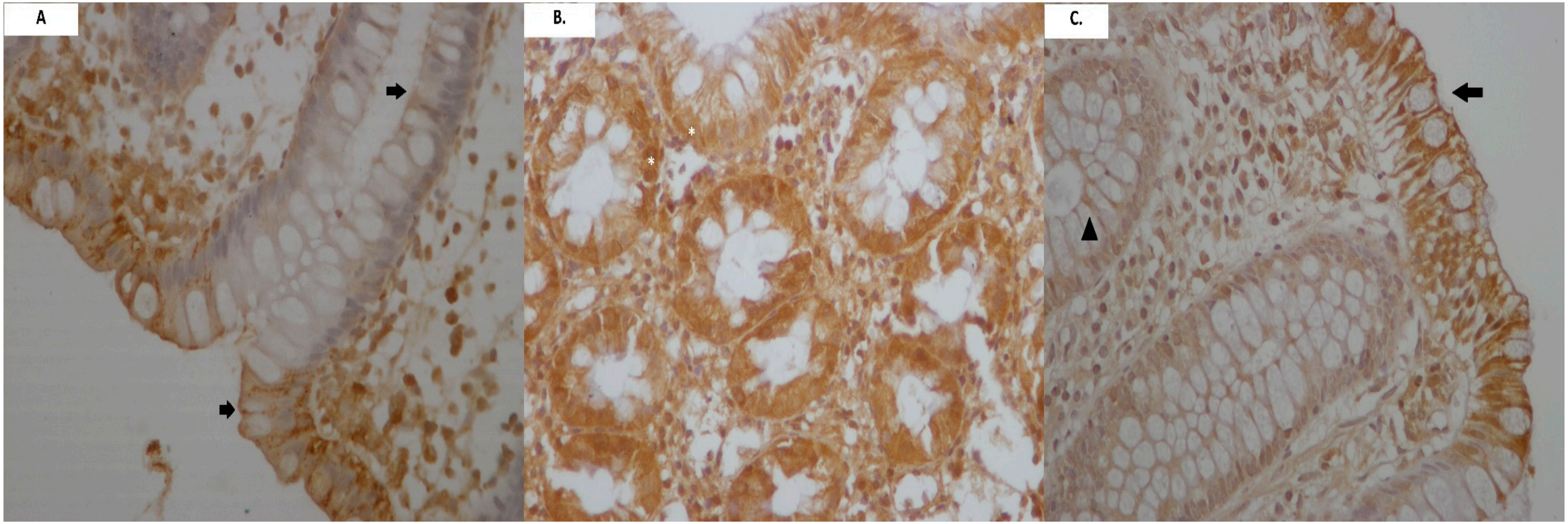

(A) Healthy control mucosa showing predominantly membranous claudin-1 staining in surface and crypt epithelial cells (arrows), consistent with a regular staining pattern. **(B)** Active Crohn's disease demonstrating increased claudin-1 immunoreactivity, predominantly within the crypt epithelium, together with an irregular staining pattern characterized by prominent cytoplasmic redistribution (asterisks). **(C)** Follow-up biopsy after therapy demonstrating reduced claudin-1 expression in the crypt epithelium (arrowhead) and partial re-localization to the membrane with persistent cytoplasmic staining (arrow). Original magnification: A and C, ×400; B, ×300.

Supplementary Figure S3. Immunohistochemical staining of occludin in intestinal mucosa in ulcerative colitis.

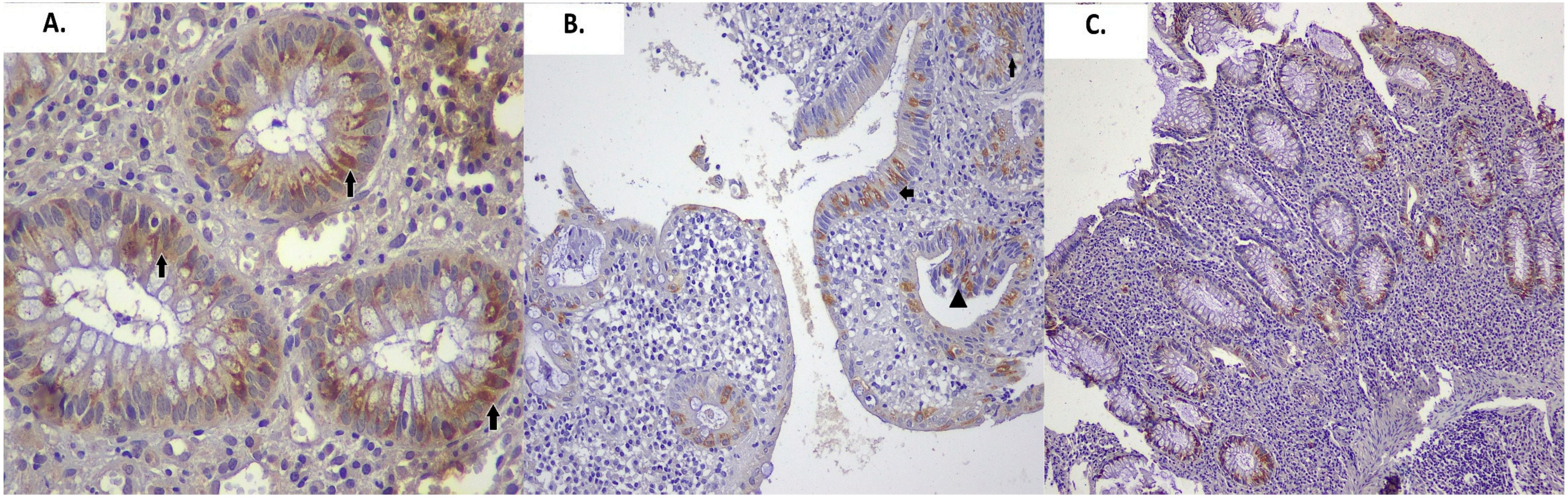

(A) Active ulcerative colitis at diagnosis demonstrating increased occludin immunoreactivity with a predominantly cytoplasmic, irregular staining pattern within the crypt epithelium (arrows). (B) Active ulcerative colitis with dense lamina propria inflammation and crypt abscess formation (arrowhead), showing irregular occludin staining characterized by patchy membranous labeling and cytoplasmic redistribution (arrows), predominantly within the crypt epithelium. (C) Follow-up biopsy under therapy demonstrating residual architectural distortion with reduced occludin expression and partial restoration of membranous localization in the crypt epithelium. Focal cytoplasmic staining persists in scattered epithelial cells. Original magnification: A, ×400; B, ×200; C, ×100.

Supplementary Figure S4. Immunohistochemical staining of claudin-1 in intestinal mucosa in ulcerative colitis.

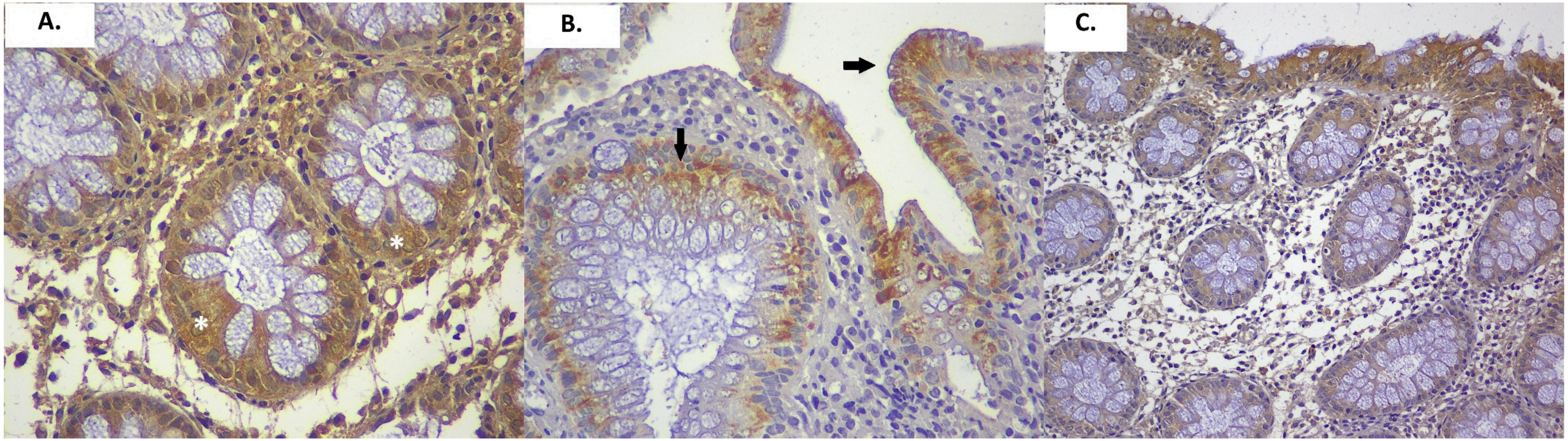

(A) Ulcerative colitis at diagnosis showing upregulated claudin-1 expression in crypt epithelial cells, with combined membranous staining and marked cytoplasmic redistribution (asterisks). (B) Active ulcerative colitis showing upregulated claudin-1 expression in the crypt epithelium with prominent cytoplasmic redistribution (downward arrow), consistent with an irregular staining phenotype, while the surface epithelium (horizontal arrow) retains retains partial membranous and moderate cytoplasmic staining. **(C)** Post-treatment ulcerative colitis showing reduced claudin-1 expression with decreased cytoplasmic staining and partial re-emergence of membranous labeling. Original magnification: A and B, ×400; C, ×200.
